# Force Control of a Haptic Flexible-Link Antenna Based on a Lumped-Mass Model

**DOI:** 10.3390/biomimetics9070414

**Published:** 2024-07-07

**Authors:** María Isabel Haro-Olmo, Luis Mérida-Calvo, Daniel Feliu-Talegón, Vicente Feliu-Batlle

**Affiliations:** 1 Instituto de Investigaciones Energéticas y Aplicaciones Industriales, Universidad de Castilla-La Mancha, 13005 Ciudad Real, Spain; misabel.haro@alu.uclm.es (M.I.H.-O.); luis.merida@alu.uclm.es (L.M.-C.); 2 Department of Mechanical and Nuclear Engineering, Khalifa University of Science and Technology, Abu Dhabi P.O. Box 127788, United Arab Emirates; daniel.talegon@ku.ac.ae; 3 Escuela Técnica Superior de Ingeniería Industrial, Universidad de Castilla-La Mancha, 13071 Ciudad Real, Spain

**Keywords:** force control, contact point flexible beam, impact detection, sensing antenna, robotic sensor, tactile sensing, robot navigation

## Abstract

Haptic organs are common in nature and help animals to navigate environments where vision is not possible. Insects often use slender, lightweight, and flexible links as sensing antennae. These antennae have a muscle-endowed base that changes their orientation and an organ that senses the applied force and moment, enabling active sensing. Sensing antennae detect obstacles through contact during motion and even recognize objects. They can also push obstacles. In all these tasks, force control of the antenna is crucial. The objective of our research is to develop a haptic robotic system based on a sensing antenna, consisting of a very lightweight and slender flexible rod. In this context, the work presented here focuses on the force control of this device. To achieve this, (a) we develop a dynamic model of the antenna that moves under gravity and maintains point contact with an object, based on lumped-mass discretization of the rod; (b) we prove the robust stability property of the closed-loop system using the Routh stability criterion; and (c) based on this property, we design a robust force control system that performs efficiently regardless of the contact point with the object. We built a mechanical device replicating this sensing organ. It is a flexible link connected at one end to a 3D force–torque sensor, which is attached to a mechanical structure with two DC motors, providing azimuthal and elevation movements to the antenna. Our experiments in contact situations demonstrate the effectiveness of our control method.

## 1. Introduction

**Figure 1:**
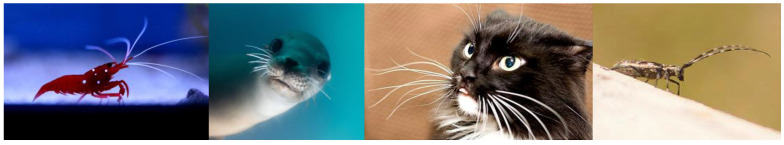
Haptic sensors of the antenna and whiskers types in nature.

Haptics is the technology of touch. In recent years, there has been increasing interest in developing integrated sensory systems, particularly those involving various tactile sensors [1]. Multiple applications require integrated systems, such as machine assemblies for precise positioning, impact protection, navigation, etc. Tactile/touch sensing is essential for developing human–machine interfaces and electronic skins in areas such as automation, security, and medical care [2]. Tactile sensors were first explored in the early 1990s, for example, in the work of Russell [3]. Since then, natural tactile sensors, including whiskers and antennae, have been investigated, e.g., [4]. Several attempts have been made to build biomimetic active sensory applications, also known as vibrational systems. Mammal and insect sensing (see Figure 1) has inspired multiple engineering applications, such as the whisker-based texture discrimination presented in [5]. The less frequent use of tactile sensing may be partly attributed to its complex and distributed nature. Issues such as sensor placement, robustness, and wiring complexity, among others, make its effective utilization challenging.

Previous works have provided evidence that artificial vibrissal systems can compute estimates of distance and shape [6,7,8] and can distinguish between textures with different spatial frequencies [9,10]. These results demonstrate the potential for vibrissal sensors as effective devices for tactile object recognition.

**Figure 2:**
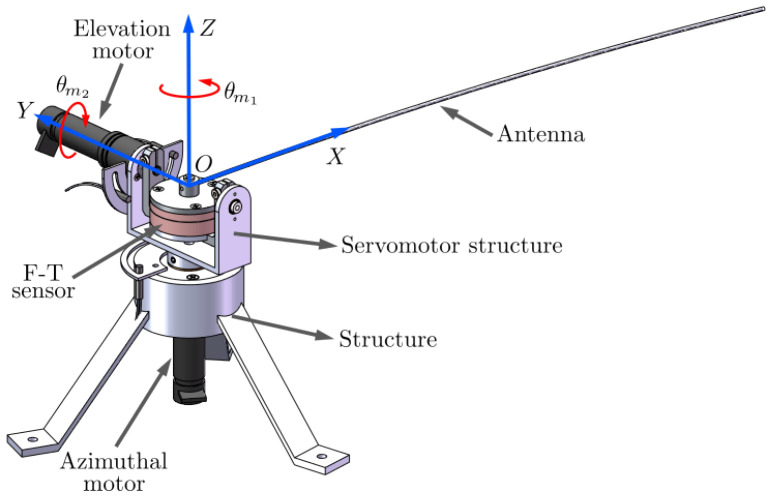
Robot Setup.

Sensors used in these systems include electret microphones [11], resistive arrays [12], strain gauges [7], piezoelectric sensors [9], and magnetic Hall-effect sensors [9,13]. Each of these technologies has its advantages and disadvantages. In the last two decades, a robust and compact sensor device called the “sensing antenna” has been proposed, efficiently addressing some of the aforementioned problems. This active sensor consists of a flexible beam moved by servo-controlled motors and a load cell placed between the beam and the motors. An example of this device is shown in Figure 2.

The sensing antenna replicates the touch sensors found in some animals and employs an active sensing strategy. The servomotor system moves the beam back and forth until it hits an object. At this instant, information from the motor angles combined with force and torque measurements allows us to calculate the positions of the hit points, which represent valuable information about the object surface. Using this device, a 3D map of the object’s surface, which enables recognition, can be obtained [13]. Recognition is carried out using techniques that combines information on partial views to gather comprehensive information about the object, e.g., [14,15].

Two strategies can be applied to obtain such 3D maps. The first strategy involves continuously moving the beam back and forth to hit the object at different points, determining their 3D coordinates and producing a map of the object surface. This strategy is used by some insects that employ their antennae for this purpose (e.g., [16,17]). The second strategy involves sliding the beam across the object while exerting a controlled force on the surface of the object to maintain contact, collecting 3D coordinates of points on the object’s surface during this movement. This strategy is utilized by some mammals with whiskers as sensors (e.g., [18,19]). Both strategies can be implemented using the aforementioned sensing antennae and require precise control of the force exerted by the antenna on the object (e.g., [20]). Additionally, if the object or obstacle needs to be removed, force control is also necessary.

However, multiple constraints limit the performance of these devices, such as their flexible beam length, light weight, and flexibility. These characteristics make the dynamic behavior of these antennae exhibit an infinite number of vibration modes, resulting in dynamic models of infinite dimension [21]. This complexity makes it very difficult to accurately control the position of these devices or achieve precise force control. If the control system in charge of moving the motors does not consider these dynamics, i.e., the beam elasticity, residual vibrations appear that prevent the accurate and fast achievement of the desired pushing force on the object being searched or moved. Moreover, permanent collisions with the object could occur, where the antenna continuously moves back and forth as it collides with the object. These phenomena would cause delays in the recognition process and diminish the quality of object surface estimates, and therefore reduce the efficiency of the device’s functioning.

It is well known that the amplitudes of the vibration modes of flexible beams diminish as the frequencies of the modes increase. This allows us to truncate their infinite-order models to finite-order models (e.g., [22]) that usually include as many as three or four vibration modes, which yield accurate approximations of the antennae dynamics. This truncation can be applied to the dynamic model obtained using an assumed-modes modeling approach (e.g., [23]), or directly to the mechanism by assuming lumped masses (e.g., [24]).

Assumed modes (e.g., [23]) have already been used to model the contact dynamics of a beam (or flexible antenna) [25] in the horizontal plane, but never in the vertical plane, under the effect of gravity. Moreover, these models are complex and pose difficulties in designing robust control systems for the contact force. Lumped mass models have been developed for the free rotation movements of flexible beams in the horizontal plane, in attitude movements where gravity affects the dynamics [26], or in a two-degrees-of-freedom single flexible-link antenna [27]. Robust force control at the tip of the beam has been addressed using a lumped-mass model with a single lumped mass at the tip in [28] in the case of having a horizontal degree of freedom. However, a lumped-mass model has never been used to model the dynamics of the contact situation in which a flexible beam pushes an object at an intermediate point along its link. This requires the use of several lumped masses.

The control of the force at the tip of a single flexible link that rotates on an horizontal plane through one of its ends was studied in [29], assuming a distributed mass link. The experiments showed that direct force feedback from a sensor placed at the tip could not ensure closed-loop stability. Stable tip contact control of a distributed mass link, where a switching transition occurred between the unconstrained and constrained environments, was achieved by [30] using a PD controller that provides feedback from hub measurements. This yielded a control system robust to the mechanical impedance of the contacted object, but could not achieve force control. To increase the stability of the tip force control, some works have redefined the force output to be fed back, e.g., [31,32]. In [33], the tip-contact-force control of a constrained single-link flexible arm was performed, overcoming the non-minimum phase nature of the system by defining a new input and generating a virtual contact-force output through a parallel compensator. It was proven that the transfer function from the new input to the virtual contact-force output was minimum-phase and stable. Ref. [34] also addresses tip-contact-force control of a one-link flexible arm interacting with a rigid environment. To achieve contact-force control, a boundary controller was proposed based on an infinite-dimensional dynamic model. The contact-force control and vibration suppression problem for a constrained one-link flexible manipulator with an unknown control direction and a time-varying actuator fault was studied in [35]. Finally, we again mention [28], where fractional-order control was implemented in a massless link with a tip payload, damping rebounds and ensuring robust stability to the mechanical impedance of the contacted object.

All these works focus on the force control of a flexible link interacting with the environment, considering that contact is made at the tip. To the best of our knowledge, the force control at an intermediate point of a flexible beam in an elevation rotation movement has never been addressed, either using an assumed-modes model or a lumped-mass model. The objectives of the present research are as follows: (1) to establish a model of the dynamics of a flexible beam contacting an object at one of its intermediate points in a rotational elevation movement based on multiple lumped masses, and (2) based on that model, to define a control system that exerts a precise pushing force on an object. Both objectives represent the contributions of this paper and have never been previously addressed.

This paper is organized is as follows. After this Introduction, Section 2 presents our experimental setup of a flexible-link antenna. Section 3 develops the dynamic model based on lumped masses. Section 4 fits this model to the lowest-frequency mode obtained from an assumed-mass model. Section 5 obtains the transfer functions of our prototype, and Section 6 derives a robust control system based on these functions. Section 7 presents our experimental results, and Section 8 offers our conclusions.

## 2. Experimental Setup

The experimental prototype is a two-degrees-of-freedom (2DOF) robotic system with a single flexible link, which is used as a sensing antenna in haptics applications. A detailed 3D representation is shown in Figure 2. Its design was developed by our group in previous works [36], where it has been employed as a tactile sensor to detect objects in its surroundings.

The flexible link, also referred to as antenna, is a lightweight, slender carbon-fiber rod with a circular cross-section. It is fixed at one of its ends (the base), while the other end moves freely (the tip). The antenna is attached at the base to a six-axis ATI FTD-MINI40 force–torque (F-T) sensor, which measures the Cartesian reacting forces and torques generated by the link. The signals are acquired through gauges located inside the sensor, which are multiplexed and amplified to send the information regarding forces and torques to a data acquisition card (DAQ). Holding the sensor and the antenna there is the servomotor structure, which is driven by two Harmonic Drive PMA-5A direct-current (DC) mini-servo actuator motor sets, featuring zero-backlash 1:100 reduction gears. One servomotor rotates the system with azimuthal movements (horizontal plane), while the other rotates it with elevation movements (vertical plane). These DC motors have incremental optical encoders that measure the angular position of the motors, $\theta_{m_{1}}$ and $\theta_{m_{2}}$, corresponding to the azimuthal and elevation joints, respectively. Additionally, a stainless-steel structure holds all this equipment and fixes the system to a flat surface with three legs to ensure perfect stability. The robot is connected to a PC through data acquisition cards. The data acquisition and control algorithms were programmed using LabVIEW NXG 5.1 with a sampling time of $T_{s}=1$ ms. All work related to data analysis and representation was carried out using MATLAB 8.2.0.29 (R2013b).

## 3. Dynamic Model

This section focuses on the modeling of a flexible beam connected to a motor. This model enables us to characterize the active sensor mentioned earlier, which moves in a vertical rotation back and forth within a plane until it makes contact with an object. Thus, the effect of gravity is considered. For this study, we assume that the interaction between the structure and the environment occurs at a single point of contact along the beam. Additionally, we assume that the force applied by the object on the beam is perpendicular to it. This assumption neglects any slipping that may occur between the two bodies. Furthermore, the contacted object is assumed to be rigid.

The dynamic model of the system is divided into two parts to describe the behavior of the motor and the flexible beam. These subsystems are interconnected through the motor angle and the torque exerted on the motor by the beam, known as the coupling torque.

### 3.1. Beam Dynamics

The flexible beam is characterized by its length *L*, linear mass density ρ, and flexural rigidity EI. It is assumed that the beam is described by a massless link with *n* lumped masses along its length, as presented in [24]. The beam exhibits small deflections, i.e, deflection lower that 10% of *L*, allowing us to use a linear deflection model [37]. Furthermore, the internal and external friction effects of the beam are neglected.

**Figure 3:**
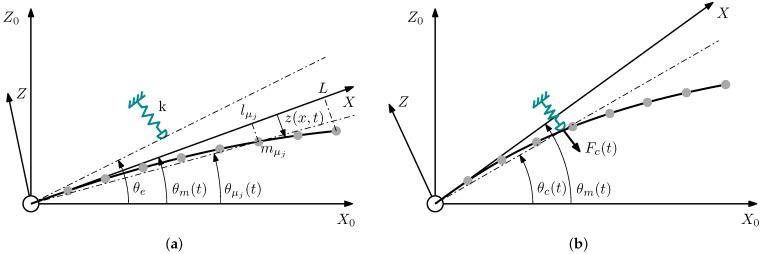
Scheme of the flexible beam (**a**) prior to contact and (**b**) after contact.

The deflection, denoted as z(x,t), is measured relative to its undeformed position, defined by the frame (X,Z). As illustrated in Figure 3, the frame (X,Z) rotates relative to a fixed frame $\left(X_{0},Z_{0}\right)$. This rotation is given by the angle of the motor $\theta_{m}\left(t\right)$. Furthermore, the lumped mass $m_{j}$ is located at distance $l_{\mu_{j}}$ and angle $\theta_{\mu_{j}}\left(t\right)$ with respect to the axis *X* and the frame $\left(X_{0},Z_{0}\right)$, respectively. It should be noted that the mass $m_{n}$ is placed at the tip of the beam, i.e., $l_{\mu_{n}}=L$.

Non-rigid contact is defined by two angles with respect to $\left(X_{0},Z_{0}\right)$: the equilibrium angle of the surface of the contacted object, $\theta_{e}$, and the angle at which the link has penetrated into the object, $\theta_{c}\left(t\right)$. If the contact is rigid, then $\theta_{c}\left(t\right)=\theta_{e}$. The contact position is defined by the distance $l_{c}$ along the *X*-axis.

Considering gravity, its direction opposes the $Z_{0}$-axis. The effect of gravity on the beam is assumed to be the force computed on the undeformed beam. This is based on the principle of superposition, which can be applied when considering small deformations [38].

Therefore, the deflection of a massless beam is given by

(1) $$EI\frac{\partial^{4}z}{\partial x^{4}}\left(x,t\right)=0$$

and is related to the angles of the system by means of

(2) $$z\left(x,t\right)=x\left(\theta \left(x,t\right)-\theta_{m}\left(t\right)\right)$$

where θ(x,t) is the angle between any point of the beam and the frame $\left(X_{0},Z_{0}\right)$.

The solution of Equation (1) is given by a piecewise function defined as

(3) $$z_{i}\left(x,t\right)=u_{i,0}\left(t\right)+u_{i,1}\left(t\right)\;\left(x-l_{i-1}\right)+u_{i,2}\left(t\right)\;{\left(x-l_{i-1}\right)}^{2}+u_{i,3}\left(t\right)\;{\left(x-l_{i-1}\right)}^{3}$$

for the interval $\left[l_{i-1},l_{i}\right]$ with i=1,2,…,N and with $l_{0}=0$ and $l_{N}=L$. Here, *N* can be either N=n or N=n+1 depending on whether contact occurs or at which position it occurs. The distance $l_{i}$ is determined by the position of either one of the lumped masses $l_{\mu_{j}}$ or the contact point $l_{c}$.

The polynomial coefficients $u_{i,j}\left(t\right)$ are obtained from the following conditions

(4) $$\begin{array}{c} z_{1}\left(0,t\right)=0;\;\frac{\partial z_{1}}{\partial x}\left(0,t\right)=0 \end{array}$$

(5) $$\begin{array}{c} \frac{\partial^{2}z_{N}}{\partial x^{2}}\left(L,t\right)=0;\;\frac{\partial^{3}z_{N}}{\partial x^{3}}\left(L,t\right)=\frac{F_{N}\left(t\right)}{EI} \end{array}$$

(6) $$\begin{array}{c} \begin{array}{c} z_{i-1}\left(l_{i-1},t\right)=z_{i}\left(l_{i-1},t\right);\;\frac{\partial z_{i-1}}{\partial x}\left(l_{i-1},t\right)=\frac{\partial z_{i}}{\partial x}\left(l_{i-1},t\right)\\ \frac{\partial^{2}z_{i-1}}{\partial x^{2}}\left(l_{i-1},t\right)=\frac{\partial^{2}z_{i}}{\partial x^{2}}\left(l_{i-1},t\right);\;\frac{\partial^{3}z_{i-1}}{\partial x^{3}}\left(l_{i-1},t\right)=\frac{\partial^{3}z_{i}}{\partial x^{3}}\left(l_{i-1},t\right)+\frac{F_{i-1}\left(t\right)}{EI} \end{array} \end{array}$$

where (4) and (5) are the boundary conditions at the joint with the motor and the tip of the beam, respectively. Equation (6) represents the continuity conditions with i=2,…,N. The force $F_{i}\left(t\right)$ is defined by

(7) $$F_{i}\left(t\right)=\left\{\begin{array}{cc} m_{i}\left(l_{i}\frac{d^{2}\theta_{i}\left(t\right)}{d^{2}t}+g\cos\left(\theta_{m}\left(t\right)\right)\right)\; & l_{i}\left(t\right)=l_{\mu_{i}}\left(t\right)\neq l_{c}\left(t\right)\\ k\left(\theta_{i}\left(t\right)-\theta_{e}\right)=-F_{c}\left(t\right)\; & l_{i}\left(t\right)=l_{c}\left(t\right)\neq l_{\mu_{i}}\left(t\right)\\ m_{i}\left(l_{i}\frac{d^{2}\theta_{i}\left(t\right)}{d^{2}t}+g\cos\left(\theta_{m}\left(t\right)\right)\right)-F_{c}\left(t\right)\; & l_{i}\left(t\right)=l_{\mu_{i}}\left(t\right)=l_{c}\left(t\right) \end{array}\right.$$

where *k* is the stiffness of the contacted object. If the contact is rigid, then *k* tends to infinity. Additionally, as previously mentioned, gravity is applied with respect to the undeformed position of the beam.

The coupling torque between the beam and the motor is given by

(8) $$\Gamma_{coup}\left(t\right)=-EI\frac{\partial^{2}z_{1}}{\partial x^{2}}\left(0,t\right)$$

Hereinafter, we work with the nondimensional model to ensure generality and applicability to any slewing flexible beam. Defining $T=\sqrt{\frac{\rho L^{4}}{EI}}$, we obtain the nondimensional time τ=t/T and frequency $\omega =\omega_{d}T$ (letting $\omega_{d}$ be the natural frequency of the flexible beam). The nondimensional spatial coordinate and deflection are χ=x/L and ζ(χ,τ)=z(x,t)/L. The forces and their positions are defined as ${ϝ}_{i}\left(\tau \right)=F_{i}\left(t\right)\frac{T^{2}}{\rho L^{2}}$ and $\lambda_{i}=l_{i}/L$, respectively, and the nondimensional torque is $\Gamma \left(\tau \right)=\Gamma \left(t\right)\frac{T^{2}}{\rho L^{3}}$. Moreover, the masses are defined as $\mu_{i}=\frac{m_{i}}{\rho L}$, gravity as $\hat{g}=g\frac{T^{2}}{L}$, and the angles as $\theta_{i}\left(\tau \right)=\theta_{i}\left(t\right)$.

Thus, the nondimensional form of Equation (1) is

(9) $$\zeta^{\prime \prime \prime \prime }\left(\chi ,\tau \right)=0$$

where, from now on, ( ˙ ) and ( ′ ) denote the derivatives with respect to the nondimensional time and spatial variables, respectively.

The relation between the deflection and the angles (2) is now

(10) $$\zeta \left(\chi ,\tau \right)=\chi \left(\theta \left(\chi ,\tau \right)-\theta_{m}\left(\tau \right)\right)$$

and the solution of the deflection (3) is

(11) $$\zeta_{i}\left(\chi ,\tau \right)=\upsilon_{i,0}\left(\tau \right)+\upsilon_{i,1}\left(\tau \right)\;\left(\chi -\lambda_{i-1}\right)+\upsilon_{i,2}\left(\tau \right)\;{\left(\chi -\lambda_{i-1}\right)}^{2}+\upsilon_{i,3}\left(\tau \right)\;{\left(\chi -\lambda_{i-1}\right)}^{3}$$

where $\upsilon_{i,0}\left(\tau \right)=u_{i,0}\left(t\right)/L$, $\upsilon_{i,1}\left(\tau \right)=u_{i,1}\left(t\right)$, $\upsilon_{i,2}\left(\tau \right)=u_{i,2}\left(t\right)L$ and $\upsilon_{i,3}\left(\tau \right)=u_{i,3}\left(t\right)L^{2}$. Furthermore, the conditions (4)–(6) and the forces (7) are

(12) $$\begin{array}{c} \zeta_{1}\left(0,\tau \right)=0;\;\zeta_{1}^{\prime }\left(0,\tau \right)=0 \end{array}$$

(13) $$\begin{array}{c} \zeta_{N}^{\prime \prime }\left(1,\tau \right)=0;\;\zeta_{N}^{\prime \prime \prime }\left(1,\tau \right)={ϝ}_{N}\left(\tau \right) \end{array}$$

(14) $$\begin{array}{c} \begin{array}{c} \zeta_{i-1}\left(\lambda_{i-1},\tau \right)=\zeta_{i}\left(\lambda_{i-1},\tau \right);\;\zeta_{i-1}^{\prime }\left(\lambda_{i-1},\tau \right)=\zeta_{i}^{\prime }\left(\lambda_{i-1},\tau \right)\\ \zeta_{i-1}^{\prime \prime }\left(\lambda_{i-1},\tau \right)=\zeta_{i}^{\prime \prime }\left(\lambda_{i-1},\tau \right);\;\zeta_{i-1}^{\prime \prime \prime }\left(\lambda_{i-1},\tau \right)=\zeta_{i}^{\prime \prime \prime }\left(\lambda_{i-1},\tau \right)+{ϝ}_{i-1}\left(\tau \right) \end{array} \end{array}$$

(15) $${ϝ}_{i}\left(\tau \right)=\left\{\begin{array}{cc} \mu_{j}\left(\lambda_{i}{\ddot{\theta }}_{i}\left(\tau \right)+\hat{g}\cos\left(\theta_{m}\left(\tau \right)\right)\right)\; & \lambda_{i}=\lambda_{\mu_{j}}\neq \lambda_{c}\\ \frac{kT^{2}}{\rho L^{2}}\left(\theta_{i}\left(\tau \right)-\theta_{e}\right)=-{ϝ}_{c}\left(\tau \right)\; & \lambda_{i}=\lambda_{c}\neq \lambda_{\mu_{j}}\\ \mu_{j}\left(\lambda_{i}{\ddot{\theta }}_{i}\left(\tau \right)+\hat{g}\cos\left(\theta_{m}\left(\tau \right)\right)\right)-{ϝ}_{c}\left(\tau \right)\; & \lambda_{i}=\lambda_{\mu_{j}}=\lambda_{c} \end{array}\right.$$

The coefficients $\upsilon_{i,j}\left(\tau \right)$ obtained by conditions (12)–(14) are presented below, while their derivation is detailed in Appendix A. For the two first coefficients, it is found that $\upsilon_{1,0}\left(\tau \right)=0$, $\upsilon_{1,1}\left(\tau \right)=0$ and

(16) $$\upsilon_{i,0}\left(\tau \right)=-\frac{1}{6}\left(\sum_{p=1}^{i-2}{ϝ}_{p}\left(\tau \right)\;\lambda_{p}^{2}\left(3\lambda_{i-1}-\lambda_{p}\right)+{ϝ}_{i-1}\left(\tau \right)\;2\lambda_{i-1}^{3}+\sum_{p=i}^{N}{ϝ}_{p}\left(\tau \right)\;\lambda_{i-1}^{2}\left(3\lambda_{p}-\lambda_{i-1}\right)\right)$$

(17) $$\upsilon_{i,1}\left(\tau \right)=-\frac{1}{2}\left(\sum_{p=1}^{i-1}{ϝ}_{p}\left(\tau \right)\;\lambda_{p}^{2}+\sum_{p=i}^{N}{ϝ}_{p}\left(\tau \right)\;\lambda_{i-1}\left(2\lambda_{p}-\lambda_{i-1}\right)\right)$$

with i=2,…,N, whereas, the others are

(18) $$\upsilon_{i,2}\left(\tau \right)=-\frac{1}{2}\sum_{p=i}^{N}{ϝ}_{p}\left(\tau \right)\;\left(\lambda_{p}-\lambda_{i-1}\right)$$

(19) $$\upsilon_{i,3}\left(\tau \right)=\frac{1}{6}\sum_{p=i}^{N}{ϝ}_{p}\left(\tau \right)$$

with i=1,…,N. Therefore, using the solution of the deflection in (10), the following equation is obtained

(20) $$-\frac{1}{6}\left(\sum_{p=1}^{i-1}{ϝ}_{p}\left(\tau \right)\;\frac{\lambda_{p}^{2}}{\lambda_{i}}\left(3\lambda_{i}-\lambda_{p}\right)+\sum_{p=i}^{N}{ϝ}_{p}\left(\tau \right)\;\lambda_{i}\left(3\lambda_{p}-\lambda_{i}\right)\right)=\theta_{i}\left(\tau \right)-\theta_{m}\left(\tau \right)$$

Furthermore, the coupling torque of Equation (8) becomes

(21) $$\Gamma_{coup}\left(\tau \right)=-\zeta_{1}^{\prime \prime }\left(0,\tau \right)=\sum_{p=1}^{N}{ϝ}_{p}\left(\tau \right)\;\lambda_{p}$$

Finally, the general solution defined in (20) can be employed to derive the dynamic equations of the beam in two cases: when the beam is freely vibrating and when it is in contact with an object.

#### 3.1.1. Free-Vibration Model

When the link vibrates freely, the forces are only due to the concentrated masses of the model, which can be expressed as ${ϝ}_{i}\left(\tau \right)=\mu_{j}\left(\lambda_{\mu_{i}}{\ddot{\theta }}_{i}\left(\tau \right)+\hat{g}\cos\left(\theta_{m}\left(\tau \right)\right)\right)$. Therefore, for this case, the number of intervals into which the displacement ζ(χ,τ) is divided is equal to the number of masses, N=n, and the distances $\lambda_{i}$ and angles $\theta_{i}\left(\tau \right)$ are $\lambda_{\mu_{j}}$ and $\theta_{\mu_{j}}$ (with i,j=1,…,n).

Thus, Equation (20) and the coupling torque of (21) become

(22) $$\begin{array}{c} -\frac{1}{6}\left(\sum_{p=1}^{j-1}\mu_{p}\left({\ddot{\theta }}_{\mu_{p}}\left(\tau \right)+\frac{1}{\lambda_{\mu_{p}}}\hat{g}\cos\left(\theta_{m}\left(\tau \right)\right)\right)\frac{\lambda_{\mu_{p}}^{3}}{\lambda_{\mu_{j}}}\left(3\lambda_{\mu_{j}}-\lambda_{\mu_{p}}\right)\right.\\ \left.+\sum_{p=j}^{n}\mu_{p}\left({\ddot{\theta }}_{\mu_{p}}\left(\tau \right)+\frac{1}{\lambda_{\mu_{p}}}\hat{g}\cos\left(\theta_{m}\left(\tau \right)\right)\right)\lambda_{\mu_{j}}\lambda_{\mu_{p}}\left(3\lambda_{\mu_{p}}-\lambda_{\mu_{j}}\right)\right)=\theta_{\mu_{j}}\left(\tau \right)-\theta_{m}\left(\tau \right) \end{array}$$

(23) $$\Gamma_{coup}\left(\tau \right)=\sum_{p=1}^{n}\mu_{p}\left({\ddot{\theta }}_{\mu_{p}}\left(\tau \right)+\frac{1}{\lambda_{\mu_{p}}}\hat{g}\cos\left(\theta_{m}\left(\tau \right)\right)\right)\;\lambda_{\mu_{p}}^{2}$$

These equations are expressed in a compact form as

(24) $$-\frac{1}{6}HM\left({\ddot{\theta }}_{\mu }+\Lambda_{1}\hat{g}\cos\left(\theta_{m}\left(\tau \right)\right)\right)=\theta_{\mu }-1_{n}\;\theta_{m}\left(\tau \right)$$

(25) $$\Gamma_{coup}\left(\tau \right)=\Lambda_{2}M\left({\ddot{\theta }}_{\mu }+\Lambda_{1}\hat{g}\cos\left(\theta_{m}\left(\tau \right)\right)\right)$$

where $1_{n}$ is a vector of ones belonging to ${ℜ}^{n\times 1}$, and

(26) $$\theta_{\mu }={\left[\begin{array}{cccc} \theta_{\mu_{1}}\left(\tau \right) & \theta_{\mu_{2}}\left(\tau \right) & \cdots & \theta_{\mu_{n}}\left(\tau \right) \end{array}\right]}^{⊺}\in {ℜ}^{n\times 1}$$

(27) $$M=diag\left(\mu_{1},\;\mu_{2},\;\ldots ,\;\mu_{n}\right)\in {ℜ}^{n\times n}$$

(28) $$H=\left[\begin{array}{cccc} h_{1,1} & h_{1,2} & \cdots & h_{1,n}\\ h_{2,1} & h_{2,2} & \cdots & h_{2,n}\\ \vdots & \vdots & \ddots & \vdots \\ h_{n,1} & h_{n,2} & \cdots & h_{n,n} \end{array}\right]\in {ℜ}^{n\times n};\;h_{j,p}=\left\{\begin{array}{cc} 2\lambda_{\mu_{j}}^{3}\; & p=j\\ \frac{\lambda_{\mu_{p}}^{3}}{\lambda_{\mu_{j}}}\left(3\lambda_{\mu_{j}}-\lambda_{\mu_{p}}\right)\; & p<j\\ \lambda_{\mu_{j}}\lambda_{\mu_{p}}\left(3\lambda_{\mu_{p}}-\lambda_{\mu_{j}}\right)\; & p>j \end{array}\right.$$

(29) $$\Lambda_{1}={\left[\begin{array}{cccc} \frac{1}{\lambda_{\mu_{1}}} & \frac{1}{\lambda_{\mu_{2}}} & \cdots & \frac{1}{\lambda_{\mu_{n}}} \end{array}\right]}^{⊺}\in {ℜ}^{n\times 1}$$

(30) $$\Lambda_{2}=\left[\begin{array}{cccc} \lambda_{\mu_{1}}^{2} & \lambda_{\mu_{2}}^{2} & \cdots & \lambda_{\mu_{n}}^{2} \end{array}\right]\in {ℜ}^{1\times n}$$

Finally, by manipulating the above equations, we obtain a dynamic model for the case of free vibrations:

(31) $${\ddot{\theta }}_{\mu }+6{\left(HM\right)}^{-1}\theta_{\mu }=6{\left(HM\right)}^{-1}1_{n}\;\theta_{m}\left(\tau \right)-\Lambda_{1}\hat{g}\cos\left(\theta_{m}\left(\tau \right)\right)$$

(32) $$\Gamma_{coup}\left(\tau \right)=-6\Lambda_{2}H^{-1}\left(\theta_{\mu }-1_{n}\;\theta_{m}\left(\tau \right)\right)$$

#### 3.1.2. Contact Model

Upon establishing contact, the beam oscillates around the position of the object due to the assumption of small displacements. For this reason, the following incremental angles are defined:

(33) $$\Delta \theta_{i}\left(\tau \right)=\theta_{i}\left(\tau \right)-\theta_{e};\;\Delta \theta_{\mu_{j}}\left(\tau \right)=\theta_{\mu_{j}}\left(\tau \right)-\theta_{e};\;\Delta \theta_{c}\left(\tau \right)=\theta_{c}\left(\tau \right)-\theta_{e}$$

Furthermore, the shape and dimensions of the model will depend on the relative position between the contact and the masses. Contact may occur at an intermediate position between two masses or coincide with one of them.

In the case of contact between masses, the displacement is divided into N=n+1 intervals. The distances $\lambda_{i}$ and angles $\theta_{i}\left(\tau \right)$ (with i=1,…,N) take the values of $\lambda_{\mu_{j}}$ and $\theta_{\mu_{j}}$ (with j=1,…,n) or $\lambda_{c}$ and $\theta_{c}$. The equations derived from Equation (20) are

(34) $$\begin{array}{c} -\frac{1}{6}\left(\sum_{p=1}^{j-1}\mu_{p}\left(\Delta {\ddot{\theta }}_{\mu_{p}}\left(\tau \right)+\frac{1}{\lambda_{\mu_{p}}}\hat{g}\cos\left(\theta_{m}\left(\tau \right)\right)\right)\frac{\lambda_{\mu_{p}}^{3}}{\lambda_{\mu_{j}}}\left(3\lambda_{\mu_{j}}-\lambda_{\mu_{p}}\right)-\frac{{ϝ}_{c}\left(\tau \right)}{\lambda_{c}}\;\lambda_{\mu_{j}}\lambda_{c}\left(3\lambda_{c}-\lambda_{\mu_{j}}\right)\right.\\ \left.+\sum_{p=j}^{n}\mu_{p}\left(\Delta {\ddot{\theta }}_{\mu_{p}}\left(\tau \right)+\frac{1}{\lambda_{\mu_{p}}}\hat{g}\cos\left(\theta_{m}\left(\tau \right)\right)\right)\lambda_{\mu_{j}}\lambda_{\mu_{p}}\left(3\lambda_{\mu_{p}}-\lambda_{\mu_{j}}\right)\right)=\Delta \theta_{\mu_{j}}\left(\tau \right)-\Delta \theta_{m}\left(\tau \right) \end{array}$$

when $\lambda_{i}=\lambda_{\mu_{j}}<\lambda_{c}<\lambda_{\mu_{j+1}}$,

(35) $$\begin{array}{c} -\frac{1}{6}\left(\sum_{p=1}^{j-1}\mu_{p}\left(\Delta {\ddot{\theta }}_{\mu_{p}}\left(\tau \right)+\frac{1}{\lambda_{\mu_{p}}}\hat{g}\cos\left(\theta_{m}\left(\tau \right)\right)\right)\frac{\lambda_{\mu_{p}}^{3}}{\lambda_{c}}\left(3\lambda_{c}-\lambda_{\mu_{p}}\right)-\frac{{ϝ}_{c}\left(\tau \right)}{\lambda_{c}}\;2\lambda_{c}^{3}\right.\\ \left.+\sum_{p=j}^{n}\mu_{p}\left(\Delta {\ddot{\theta }}_{\mu_{p}}\left(\tau \right)+\frac{1}{\lambda_{\mu_{p}}}\hat{g}\cos\left(\theta_{m}\left(\tau \right)\right)\right)\lambda_{c}\lambda_{\mu_{p}}\left(3\lambda_{\mu_{p}}-\lambda_{c}\right)\right)=\Delta \theta_{c}\left(\tau \right)-\Delta \theta_{m}\left(\tau \right) \end{array}$$

when $\lambda_{\mu_{j-1}}<\lambda_{i}=\lambda_{c}<\lambda_{\mu_{j}}$, and

(36) $$\begin{array}{c} -\frac{1}{6}\left(\sum_{p=1}^{j-1}\mu_{p}\left(\Delta {\ddot{\theta }}_{\mu_{p}}\left(\tau \right)+\frac{1}{\lambda_{\mu_{p}}}\hat{g}\cos\left(\theta_{m}\left(\tau \right)\right)\right)\frac{\lambda_{\mu_{p}}^{3}}{\lambda_{\mu_{j}}}\left(3\lambda_{\mu_{j}}-\lambda_{\mu_{p}}\right)-\frac{{ϝ}_{c}\left(\tau \right)}{\lambda_{c}}\;\frac{\lambda_{c}^{3}}{\lambda_{\mu_{j}}}\left(3\lambda_{\mu_{j}}-\lambda_{c}\right)\right.\\ \left.+\sum_{p=j}^{n}\mu_{p}\left(\Delta {\ddot{\theta }}_{\mu_{p}}\left(\tau \right)+\frac{1}{\lambda_{\mu_{p}}}\hat{g}\cos\left(\theta_{m}\left(\tau \right)\right)\right)\lambda_{\mu_{j}}\lambda_{\mu_{p}}\left(3\lambda_{\mu_{p}}-\lambda_{\mu_{j}}\right)\right)=\Delta \theta_{\mu_{j}}\left(\tau \right)-\Delta \theta_{m}\left(\tau \right) \end{array}$$

when $\lambda_{\mu_{j-1}}<\lambda_{c}<\lambda_{i}=\lambda_{\mu_{j}}$.

The coupling torque (21) becomes

(37) $$\Gamma_{coup}\left(\tau \right)=-\frac{{ϝ}_{c}\left(\tau \right)}{\lambda_{c}}\lambda_{c}^{2}+\sum_{p=1}^{n}\mu_{p}\left(\Delta {\ddot{\theta }}_{\mu_{p}}\left(\tau \right)+\frac{1}{\lambda_{\mu_{p}}}\hat{g}\cos\left(\theta_{m}\left(\tau \right)\right)\right)\;\lambda_{\mu_{p}}^{2}$$

The system of equations can be expressed in a more concise form as follows:

(38) $$-\frac{1}{6}\left[\begin{array}{cc} H & H_{c1}\\ H_{c2} & 2\lambda_{c}^{3} \end{array}\right]\left[\begin{array}{cc} M & 0_{n}\\ 0_{n}^{⊺} & 1 \end{array}\right]\left[\begin{array}{c} \Delta {\ddot{\theta }}_{\mu }+\Lambda_{1}\hat{g}\cos\left(\theta_{m}\left(\tau \right)\right)\\ -\frac{{ϝ}_{c}\left(\tau \right)}{\lambda_{c}} \end{array}\right]=\left[\begin{array}{c} \Delta \theta_{\mu }\\ \Delta \theta_{c}\left(\tau \right) \end{array}\right]-1_{n+1}\;\Delta \theta_{m}\left(\tau \right)$$

(39) $$\Gamma_{coup}\left(\tau \right)=\left[\begin{array}{cc} \Lambda_{2} & \lambda_{c}^{2} \end{array}\right]\left[\begin{array}{cc} M & 0_{n}\\ 0_{n}^{⊺} & 1 \end{array}\right]\left[\begin{array}{c} \Delta {\ddot{\theta }}_{\mu }+\Lambda_{1}\hat{g}\cos\left(\theta_{m}\left(\tau \right)\right)\\ -\frac{{ϝ}_{c}\left(\tau \right)}{\lambda_{c}} \end{array}\right]$$

where $0_{n}$ is a vector of zeros in ${ℜ}^{n\times 1}$; $1_{n+1}$ is a vector of ones in ${ℜ}^{n+1\times 1}$; and M, H, $\Lambda_{1}$, and $\Lambda_{2}$ are the same as in (27)–(30) and

(40) $$\Delta \theta_{\mu }={\left[\begin{array}{cccc} \Delta \theta_{\mu_{1}}\left(\tau \right) & \Delta \theta_{\mu_{2}}\left(\tau \right) & \cdots & \Delta \theta_{\mu_{n}}\left(\tau \right) \end{array}\right]}^{⊺}\in {ℜ}^{n\times 1}$$

(41) $$H_{c1}={\left[\begin{array}{cccc} h_{1,N} & h_{2,N} & \cdots & h_{n,N} \end{array}\right]}^{⊺}\in {ℜ}^{n\times 1};\;h_{j,N}=\left\{\begin{array}{cc} \frac{\lambda_{c}^{3}}{\lambda_{\mu_{j}}}\left(3\lambda_{\mu_{j}}-\lambda_{c}\right)\; & \lambda_{c}<\lambda_{\mu_{j}}\\ \lambda_{\mu_{j}}\lambda_{c}\left(3\lambda_{c}-\lambda_{\mu_{j}}\right)\; & \lambda_{c}>\lambda_{\mu_{j}} \end{array}\right.$$

(42) $$H_{c2}=\left[\begin{array}{cccc} h_{N,1} & h_{N,2} & \cdots & h_{N,n} \end{array}\right]\in {ℜ}^{1\times n};\;h_{N,j}=\left\{\begin{array}{cc} \frac{\lambda_{\mu_{j}}^{3}}{\lambda_{c}}\left(3\lambda_{c}-\lambda_{\mu_{j}}\right)\; & \lambda_{c}<\lambda_{\mu_{j}}\\ \lambda_{c}\lambda_{\mu_{j}}\left(3\lambda_{\mu_{j}}-\lambda_{c}\right)\; & \lambda_{c}>\lambda_{\mu_{j}} \end{array}\right.$$

After calculating Equations (38) and (39), we obtain the following dynamic model:

(43) $$\Delta {\ddot{\theta }}_{\mu }+R\Delta \theta_{\mu }-\frac{1}{2\lambda_{c}^{3}}RH_{c1}\Delta \theta_{c}\left(\tau \right)=R\left(1_{n}+\frac{1}{2\lambda_{c}^{3}}H_{c1}\right)\Delta \theta_{m}\left(\tau \right)-\Lambda_{1}\hat{g}\cos\left(\theta_{m}\left(\tau \right)\right)$$

(44) $$-\frac{{ϝ}_{c}\left(\tau \right)}{\lambda_{c}}+\frac{6}{2\lambda_{c}^{3}}\Delta \theta_{c}\left(\tau \right)+\frac{1}{2\lambda_{c}^{3}}H_{c2}M\Delta {\ddot{\theta }}_{\mu }=\frac{6}{2\lambda_{c}^{3}}\Delta \theta_{m}\left(\tau \right)-\frac{1}{2\lambda_{c}^{3}}H_{c2}M\Lambda_{1}\hat{g}\cos\left(\theta_{m}\left(\tau \right)\right)$$

where

(45) $$R=6M^{-1}{\left(H-\frac{1}{2\lambda_{c}^{3}}H_{c1}H_{c2}\right)}^{-1}$$

and

(46) $$\Gamma_{coup}\left(\tau \right)=-6\left[\begin{array}{cc} \Lambda_{2} & \lambda_{c}^{2} \end{array}\right]{\left[\begin{array}{cc} H & H_{c1}\\ H_{c2} & 2\lambda_{c}^{3} \end{array}\right]}^{-1}\left(\left[\begin{array}{c} \Delta \theta_{\mu }\\ \Delta \theta_{c}\left(\tau \right) \end{array}\right]-1_{n+1}\;\Delta \theta_{m}\left(\tau \right)\right)$$

By assuming that the contact is rigid, i.e., $\Delta \theta_{c}\left(\tau \right)=0$, we can derive that the Equation (43) is

(47) $$\Delta {\ddot{\theta }}_{\mu }+R\Delta \theta_{\mu }=R\left(1_{n}+\frac{1}{2\lambda_{c}^{3}}H_{c1}\right)\Delta \theta_{m}\left(\tau \right)-\Lambda_{1}\hat{g}\cos\left(\theta_{m}\left(\tau \right)\right),$$

the coupling torque (46) is

(48) $$\Gamma_{coup}\left(\tau \right)=-\left(\Lambda_{2}-\frac{1}{2\lambda_{c}}H_{c2}\right)MR\left(\Delta \theta_{\mu }-\left(1_{n}+\frac{1}{2\lambda_{c}^{3}}H_{c1}\right)\Delta \theta_{m}\left(\tau \right)\right)+\frac{6}{2\lambda_{c}}\Delta \theta_{m}\left(\tau \right)$$

and the contact force can be obtained from (44) as

(49) $${ϝ}_{c}\left(\tau \right)=-\frac{1}{2\lambda_{c}^{2}}H_{c2}MR\left(\Delta \theta_{\mu }-\left(1_{n}+\frac{1}{2\lambda_{c}^{3}}H_{c1}\right)\Delta \theta_{m}\left(\tau \right)\right)-\frac{6}{2\lambda_{c}^{2}}\Delta \theta_{m}\left(\tau \right)$$

On the other hand, when the contact occurs in the position of one of the masses, the displacement is divided into N=n intervals and the equations derived from Equation (20) are

(50) $$\begin{array}{c} -\frac{1}{6}\left(\sum_{p=1}^{j-1}\mu_{p}\left(\Delta {\ddot{\theta }}_{\mu_{p}}\left(\tau \right)+\frac{1}{\lambda_{\mu_{p}}}\hat{g}\cos\left(\theta_{m}\left(\tau \right)\right)\right)\frac{\lambda_{\mu_{p}}^{3}}{\lambda_{\mu_{j}}}\left(3\lambda_{\mu_{j}}-\lambda_{\mu_{p}}\right)\right.\\ \left.+\sum_{p=j}^{j+q-1}\mu_{p}\left(\Delta {\ddot{\theta }}_{\mu_{p}}\left(\tau \right)+\frac{1}{\lambda_{\mu_{p}}}\hat{g}\cos\left(\theta_{m}\left(\tau \right)\right)\right)\lambda_{\mu_{j}}\lambda_{\mu_{p}}\left(3\lambda_{\mu_{p}}-\lambda_{c}\right)\right.\\ \left.+\left(\mu_{j+q}\Delta {\ddot{\theta }}_{c}\left(\tau \right)+\mu_{j+q}\frac{1}{\lambda_{c}}\hat{g}\cos\left(\theta_{m}\left(\tau \right)\right)-\frac{{ϝ}_{c}\left(\tau \right)}{\lambda_{c}}\right)\lambda_{\mu_{j}}\lambda_{c}\left(3\lambda_{c}-\lambda_{\mu_{j}}\right)\right.\\ \left.+\sum_{p=j+q+1}^{n}\mu_{p}\left(\Delta {\ddot{\theta }}_{\mu_{p}}\left(\tau \right)+\frac{1}{\lambda_{\mu_{p}}}\hat{g}\cos\left(\theta_{m}\left(\tau \right)\right)\right)\lambda_{\mu_{j}}\lambda_{\mu_{p}}\left(3\lambda_{\mu_{p}}-\lambda_{\mu_{j}}\right)\right)=\Delta \theta_{\mu_{j}}\left(\tau \right)-\Delta \theta_{m}\left(\tau \right) \end{array}$$

when $\lambda_{i}=\lambda_{\mu_{j}}<\lambda_{c}=\lambda_{\mu_{j+q}}$;

(51) $$\begin{array}{c} -\frac{1}{6}\left(\sum_{p=1}^{j-1}\mu_{p}\left(\Delta {\ddot{\theta }}_{\mu_{p}}\left(\tau \right)+\frac{1}{\lambda_{\mu_{p}}}\hat{g}\cos\left(\theta_{m}\left(\tau \right)\right)\right)\frac{\lambda_{\mu_{p}}^{3}}{\lambda_{c}}\left(3\lambda_{c}-\lambda_{\mu_{p}}\right)\right.\\ \left.+\left(\mu_{j}\Delta {\ddot{\theta }}_{c}\left(\tau \right)+\mu_{j}\frac{1}{\lambda_{c}}\hat{g}\cos\left(\theta_{m}\left(\tau \right)\right)-\frac{{ϝ}_{c}\left(\tau \right)}{\lambda_{c}}\right)2\lambda_{c}^{3}\right.\\ \left.+\sum_{p=j+1}^{n}\mu_{p}\left(\Delta {\ddot{\theta }}_{\mu_{p}}\left(\tau \right)+\frac{1}{\lambda_{\mu_{p}}}\hat{g}\cos\left(\theta_{m}\left(\tau \right)\right)\right)\lambda_{c}\lambda_{\mu_{p}}\left(3\lambda_{\mu_{p}}-\lambda_{c}\right)\right)=\Delta \theta_{c}\left(\tau \right)-\Delta \theta_{m}\left(\tau \right) \end{array}$$

when $\lambda_{i}=\lambda_{\mu_{j}}=\lambda_{c}$; and

(52) $$\begin{array}{c} -\frac{1}{6}\left(\sum_{p=1}^{j-q-1}\mu_{p}\left(\Delta {\ddot{\theta }}_{\mu_{p}}\left(\tau \right)+\frac{1}{\lambda_{\mu_{p}}}\hat{g}\cos\left(\theta_{m}\left(\tau \right)\right)\right)\frac{\lambda_{\mu_{p}}^{3}}{\lambda_{\mu_{j}}}\left(3\lambda_{\mu_{j}}-\lambda_{\mu_{p}}\right)\right.\\ \left.+\left(\mu_{j-q}\Delta {\ddot{\theta }}_{c}\left(\tau \right)+\mu_{j-q}\frac{1}{\lambda_{c}}\hat{g}\cos\left(\theta_{m}\left(\tau \right)\right)-\frac{{ϝ}_{c}\left(\tau \right)}{\lambda_{c}}\right)\frac{\lambda_{c}^{3}}{\lambda_{\mu_{j}}}\left(3\lambda_{\mu_{j}}-\lambda_{c}\right)\right.\\ \left.\sum_{p=j-q+1}^{j-1}\mu_{p}\left(\Delta {\ddot{\theta }}_{\mu_{p}}\left(\tau \right)+\frac{1}{\lambda_{\mu_{p}}}\hat{g}\cos\left(\theta_{m}\left(\tau \right)\right)\right)\frac{\lambda_{\mu_{p}}^{3}}{\lambda_{\mu_{j}}}\left(3\lambda_{\mu_{j}}-\lambda_{\mu_{p}}\right)\right.\\ \left.+\sum_{p=j}^{n}\mu_{p}\left(\Delta {\ddot{\theta }}_{\mu_{p}}\left(\tau \right)+\frac{1}{\lambda_{\mu_{p}}}\hat{g}\cos\left(\theta_{m}\left(\tau \right)\right)\right)\lambda_{\mu_{j}}\lambda_{\mu_{p}}\left(3\lambda_{\mu_{p}}-\lambda_{\mu_{j}}\right)\right)=\Delta \theta_{\mu_{j}}\left(\tau \right)-\Delta \theta_{m}\left(\tau \right) \end{array}$$

when $\lambda_{c}=\lambda_{\mu_{j-q}}<\lambda_{i}=\lambda_{\mu_{j}}$.

Here, the coupling torque is equal to (37).

Once more, the model is expressed in a concise form as follows:

(53) $$-\frac{1}{6}\left[\begin{array}{cc} \hat{H} & {\hat{H}}_{c1}\\ {\hat{H}}_{c2} & 2\lambda_{c}^{3} \end{array}\right]\left[\begin{array}{cc} \hat{M} & 0_{n-1}\\ 0_{n-1}^{⊺} & \mu_{c} \end{array}\right]\left[\begin{array}{c} \Delta {\ddot{\hat{\theta }}}_{\mu }+{\hat{\Lambda }}_{1}\hat{g}\cos\left(\theta_{m}\left(\tau \right)\right)\\ \Delta {\ddot{\theta }}_{c}\left(\tau \right)+\frac{1}{\lambda_{c}}\hat{g}\cos\left(\theta_{m}\left(\tau \right)\right)-\frac{{ϝ}_{c}\left(\tau \right)}{\mu_{c}\lambda_{c}} \end{array}\right]=\left[\begin{array}{c} \Delta {\hat{\theta }}_{\mu }\\ \Delta \theta_{c}\left(\tau \right) \end{array}\right]-1_{n}\;\Delta \Theta_{m}\left(s\right)$$

(54) $$\Gamma_{coup}\left(\tau \right)=\left[\begin{array}{cc} {\hat{\Lambda }}_{2} & \lambda_{c}^{2} \end{array}\right]\left[\begin{array}{cc} \hat{M} & 0_{n-1}\\ 0_{n-1}^{⊺} & \mu_{c} \end{array}\right]\left[\begin{array}{c} \Delta {\ddot{\hat{\theta }}}_{\mu }+{\hat{\Lambda }}_{1}\hat{g}\cos\left(\theta_{m}\left(\tau \right)\right)\\ \Delta {\ddot{\theta }}_{c}\left(\tau \right)+\frac{1}{\lambda_{c}}\hat{g}\cos\left(\theta_{m}\left(\tau \right)\right)-\frac{{ϝ}_{c}\left(\tau \right)}{\mu_{c}\lambda_{c}} \end{array}\right]$$

where the mass at which contact occurs is designated as $\mu_{c}$. The vectors $\Delta {\hat{\theta }}_{\mu }\in {ℜ}^{n-1\times 1}$, ${\hat{\Lambda }}_{1}\in {ℜ}^{n-1\times 1}$, ${\hat{\Lambda }}_{2}\in {ℜ}^{1\times n-1}$, ${\hat{H}}_{c1}\in {ℜ}^{n-1\times 1}$ and ${\hat{H}}_{c2}\in {ℜ}^{1\times n-1}$ are derived by removing from $\Delta \theta_{\mu }$, $\Lambda_{1}$, $\Lambda_{2}$, $H_{c1}$ and $H_{c2}$ the element related to the mass coincident with the contact $\mu_{c}$. Similarly, the matrices $\hat{M}\in {ℜ}^{n-1\times n-1}$ and $\hat{H}$ are derived by removing from M and H the columns and rows corresponding to the mass $\mu_{c}$.

The following dynamic model is obtained from (53) and (54)

(55) $$\Delta {\ddot{\hat{\theta }}}_{\mu }+\hat{R}\Delta {\hat{\theta }}_{\mu }-\frac{1}{2\lambda_{c}^{3}}\hat{R}{\hat{H}}_{c1}\Delta \theta_{c}\left(\tau \right)=\hat{R}\left(1_{n-1}+\frac{1}{2\lambda_{c}^{3}}{\hat{H}}_{c1}\right)\Delta \theta_{m}\left(\tau \right)-{\hat{\Lambda }}_{1}\hat{g}\cos\left(\theta_{m}\left(\tau \right)\right)$$

(56) $$\begin{array}{c} -\frac{{ϝ}_{c}\left(\tau \right)}{\lambda_{c}}+\Delta {\ddot{\theta }}_{c}\left(\tau \right)+\frac{6}{2\lambda_{c}^{3}}\Delta \theta_{c}\left(\tau \right)+\frac{1}{2\lambda_{c}^{3}}{\hat{H}}_{c2}\hat{M}\Delta {\ddot{\hat{\theta }}}_{\mu }=\frac{6}{2\lambda_{c}^{3}}\Delta \theta_{m}\left(\tau \right)\\ -\left(\frac{1}{2\lambda_{c}^{3}}{\hat{H}}_{c2}\hat{M}\Lambda_{1}+\frac{1}{\lambda_{c}}\right)\hat{g}\cos\left(\theta_{m}\left(\tau \right)\right) \end{array}$$

where

(57) $$\hat{R}=6{\hat{M}}^{-1}{\left(\hat{H}-\frac{1}{2\lambda_{c}^{3}}{\hat{H}}_{c1}{\hat{H}}_{c2}\right)}^{-1}$$

and

(58) $$\Gamma_{coup}\left(s\right)=-6\left[\begin{array}{cc} {\hat{\Lambda }}_{2} & \lambda_{c}^{2} \end{array}\right]{\left[\begin{array}{cc} \hat{H} & {\hat{H}}_{c1}\\ {\hat{H}}_{c2} & 2\lambda_{c}^{3} \end{array}\right]}^{-1}\left(\left[\begin{array}{c} \Delta {\hat{\Theta }}_{\mu }\\ \Delta \Theta_{c}\left(s\right) \end{array}\right]-1_{n}\;\Delta \Theta_{m}\left(s\right)\right)$$

Finally, by assuming rigid contact in Equations (55) and (58), we obtain

(59) $$\Delta {\ddot{\hat{\theta }}}_{\mu }+\hat{R}\Delta {\hat{\theta }}_{\mu }=\hat{R}\left(1_{n-1}+\frac{1}{2\lambda_{c}^{3}}{\hat{H}}_{c1}\right)\Delta \theta_{m}\left(\tau \right)-{\hat{\Lambda }}_{1}\hat{g}\cos\left(\theta_{m}\left(\tau \right)\right),$$

(60) $$\Gamma_{coup}\left(\tau \right)=-\left({\hat{\Lambda }}_{2}-\frac{1}{2\lambda_{c}}{\hat{H}}_{c2}\right)\hat{M}\hat{R}\left(\Delta {\hat{\theta }}_{\mu }-\left(1_{n-1}+\frac{1}{2\lambda_{c}^{3}}{\hat{H}}_{c1}\right)\Delta \theta_{m}\left(\tau \right)\right)+\frac{6}{2\lambda_{c}}\Delta \theta_{m}\left(\tau \right)$$

and Equation (56) provides us with the contact force as follows:

(61) $${ϝ}_{c}\left(\tau \right)=-\frac{1}{2\lambda_{c}^{2}}{\hat{H}}_{c2}\hat{M}\hat{R}\left(\Delta {\hat{\theta }}_{\mu }-\left(1_{n-1}+\frac{1}{2\lambda_{c}^{3}}{\hat{H}}_{c1}\right)\Delta \theta_{m}\left(\tau \right)\right)-\frac{6}{2\lambda_{c}^{2}}\Delta \theta_{m}\left(\tau \right)$$

In summary, the contact model is described by Equations (47)–(49) when the contact is between the masses, and by (59)–(61) when it coincides with the position of a mass.

*Remark*. For a given number of masses *n*, the rigid contact model has one order fewer when the contact is at one mass (Equations (59)–(61)) compared to when it is between masses (Equations (47)–(49)). Consequently, when contact occurs at one of the masses, the number of vibration frequencies is reduced by one.

### 3.2. Motors Dynamics

The behavior of the motor is described by the following equation

(62) $$J_{0}\frac{d^{2}\theta_{m}}{dt^{2}}\left(t\right)+\Gamma_{coup}\left(t\right)=\Gamma_{m}\left(t\right)$$

where $J_{0}$ is the rotational inertia of the motor and $\Gamma_{m}\left(t\right)$ is the torque produced by the actuator to move the system.

The nondimensional form of Equation (62) is

(63) $$\frac{1}{3}R_{J}{\ddot{\theta }}_{m}\left(\tau \right)+\Gamma_{coup}\left(\tau \right)=\Gamma_{m}\left(\tau \right)$$

where the nondimensional inertia of the actuator is obtained by calculating $R_{J}=\frac{J_{0}}{J_{b}}$, where $J_{b}=\frac{1}{3}\rho L^{3}$ is the rotational inertia of a distributed mass beam.

In the case of contact, where the incremental angles of Equation (33) are defined, the equation of the engine is such that

(64) $$\frac{1}{3}R_{J}\Delta {\ddot{\theta }}_{m}\left(\tau \right)+\Gamma_{coup}\left(\tau \right)=\Gamma_{m}\left(\tau \right)$$

## 4. Adjustment of the Rigid Contact Model

In this section, the parameters of the lumped-mass model described by the Equations (47)–(49) and (59)–(61) are adjusted. The aim is to ensure that the first vibration frequency of the model coincides with the frequency of a flexible beam in contact with a rigid object. This frequency, as discussed in [25], depends on the point at which the contact occurs, denoted as $\lambda_{c}$. Furthermore, the order of the model should be kept as low as possible in order to minimize the computational complexity. It is important to note that since the model is dimensionless, the results obtained here are applicable to any slewing flexible beam.

To fit the model, we start with a lower-order model where only one mass is considered, and then increase the number of masses *n* until a satisfactory result is achieved for the system frequency. The parameters to be adjusted are the masses $\mu_{j}$ and their respective distances $\lambda_{\mu_{j}}$. The conditions imposed include keeping the total mass of the link

(65) $$\sum_{j=1}^{n}\mu_{j}=1\;$$

and ensuring that the length of the link is maintained so that the last mass $\mu_{n}$ is positioned at the end of the link, i.e., $\lambda_{\mu_{n}}=1$.

*Remark*. It should be noted that the parameters of the model with one mass (n=1) are determined by the imposed conditions. Consequently, if these conditions are to be maintained, it is not possible to modify the parameters in order to obtain a better fit.

Thus, Matlab was used to adjust the above parameters with the aim of minimizing the following mean square error:

(66) $$\mathrm{MSE}=\frac{1}{N_{p}}\sum_{i=1}^{N_{p}}{\left({\bar{\omega }}_{1}\left(\lambda_{c,i}\right)-{\tilde{\omega }}_{1}\left(\lambda_{c,i}\right)\right)}^{2}$$

where ${\bar{\omega }}_{1}\left(\lambda_{c,i}\right)$ and ${\tilde{\omega }}_{1}\left(\lambda_{c,i}\right)$ represent the frequencies obtained from the model in [25] and from the lower eigenvalue of the matrix R (or $\hat{R}$), respectively. Both frequencies depend on the contact point $\lambda_{c}$. The contact point is defined in the interval (0,1] with a step of 0.01, resulting in a total number of points of $N_{p}=100$.

The smallest number of masses that achieves a satisfactory fit of the first frequency is n=3. The results for the models with a lower number of masses are presented in Appendix B.

**Figure 4:**
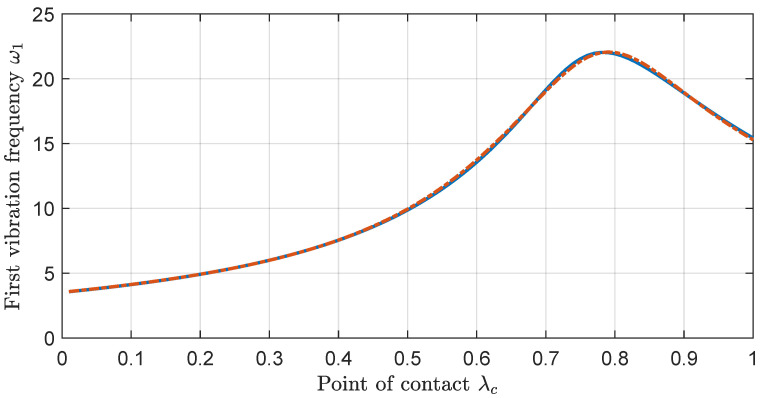
First vibration frequency as a function of contact point, where (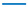) corresponds to ${\bar{\omega }}_{1}\left(\lambda_{c}\right)$, and (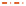) to ${\tilde{\omega }}_{1}\left(\lambda_{c}\right)$.

To achieve the fit for the three-mass model, it is considered that $\mu_{1}$ varies within the interval (0,0.99), and $\mu_{2}$ within the interval $\left(0,\mu_{1}\right)$, both with a step of 0.01. For the positions of the masses, $\lambda_{\mu_{2}}$ and $\lambda_{\mu_{1}}$ vary within the intervals (0.01,1) and $\left(0,\lambda_{2}\right)$, respectively, with a step of 0.01. Moreover, $\mu_{3}$ is defined by (65) and $\lambda_{\mu_{3}}=1$. The model with the minimum mean square error, i.e., MSE=0.008, is characterized by the parameters $\mu_{1}=0.49$, $\mu_{2}=0.41$, $\mu_{3}=0.10$, $\lambda_{\mu_{1}}=0.26$, and $\lambda_{\mu_{2}}=0.70$. The comparison between ${\bar{\omega }}_{1}\left(\lambda_{c}\right)$ and ${\tilde{\omega }}_{1}\left(\lambda_{c}\right)$ for this model is presented in Figure 4.

## 5. Flexible Beam Transfer Functions

The transfer function that relates the coupling torque to the motor angle in rigid contact is derived using the parameters from the previous section and Equations (47) and (48), or Equations (59) and (60) if the contact coincides with one of the masses. However, for the sake of clarity, the steps will be outlined for the first set of equations, but are exactly the same for the second.

As observed, the dynamic model obtained is nonlinear because gravity depends on the motor angle. Consequently, to obtain a linear model, the equations of the system are linearized around the point

(67) $$\Delta \theta_{m,0}=0$$

Thus, taking into account that $\cos\left(\theta_{m}\left(\tau \right)\right)=\cos\left(\Delta \theta_{m}\left(\tau \right)+\theta_{e}\right)$, the equilibrium point is defined by

(68) $$\Delta \theta_{\mu ,0}=-R^{-1}\Lambda_{1}\hat{g}\cos\left(\theta_{e}\right)$$

(69) $$\Gamma_{coup,0}=\left(\Lambda_{2}-\frac{1}{2\lambda_{c}}H_{c2}\right)M\Lambda_{1}\hat{g}\cos\left(\theta_{e}\right)$$

Let us define the variations from the equilibrium points as $\delta \theta_{m}\left(\tau \right)=\Delta \theta_{m}\left(\tau \right)-\Delta \theta_{m,0}$, $\delta \theta_{\mu }\left(\tau \right)=\Delta \theta_{\mu }\left(\tau \right)-\Delta \theta_{\mu ,0}$ and $\delta \Gamma_{coup}\left(\tau \right)=\Gamma_{coup}\left(\tau \right)-\Gamma_{coup,0}$. The following linearized model is obtained by using the first-order Taylor series expansion

(70) $$\delta {\ddot{\theta }}_{\mu }+R\delta \theta_{\mu }=\left(R\left(1+\frac{1}{2\lambda_{c}^{3}}H_{c1}\right)+\Lambda_{1}\hat{g}\sin\left(\theta_{e}\right)\right)\delta \theta_{m}\left(\tau \right)$$

(71) $$\delta \Gamma_{coup}\left(\tau \right)=-\left(\Lambda_{2}-\frac{1}{2\lambda_{c}}H_{c2}\right)MR\left(\delta \theta_{\mu }-\left(1+\frac{1}{2\lambda_{c}^{3}}H_{c1}\right)\delta \theta_{m}\left(\tau \right)\right)+\frac{6}{2\lambda_{c}}\delta \theta_{m}\left(\tau \right)$$

Taking the Laplace transforms of these equations and substituting $\delta \theta_{\mu }$ from (70) into (71), we obtain the transfer function between the coupling torque and the angle of the motor

(72) $$\frac{\delta \Gamma_{coup}\left(s\right)}{\delta \Theta_{m}\left(s\right)}=G\left(s,\lambda_{c},\theta_{e}\right)=G_{b}\left(s,\lambda_{c}\right)+G_{g}\left(s,\lambda_{c}\right)\;\hat{g}\sin\left(\theta_{e}\right)$$

with

(73) $$G_{b}\left(s,\lambda_{c}\right)=-\left(\Lambda_{2}-\frac{1}{2\lambda_{c}}H_{c2}\right)MR\left({\left(Is^{2}+R\right)}^{-1}R-I\right)\left(1+\frac{1}{2\lambda_{c}^{3}}H_{c1}\right)+\frac{6}{2\lambda_{c}}$$

(74) $$G_{g}\left(s,\lambda_{c}\right)=-\left(\Lambda_{2}-\frac{1}{2\lambda_{c}}H_{c2}\right)MR{\left(Is^{2}+R\right)}^{-1}\Lambda_{1}$$

Truncating the transfer function to the first mode of vibration, we obtain a function of the form

(75) $$G\left(s,\lambda_{c},\theta_{e}\right)=d\left(\lambda_{c}\right)+\frac{b\left(\lambda_{c}\right)+c\left(\lambda_{c}\right)\hat{g}\sin\left(\theta_{e}\right)}{s^{2}+a\left(\lambda_{c}\right)}$$

where the coefficients $a(\lambda_{c})$, $b(\lambda_{c})$, $c(\lambda_{c})$, and $d(\lambda_{c})$ are obtained with Matlab and are fitted by means of the functions in the Appendix C. It is important to note that the coefficient $a(\lambda_{c})$ corresponds to the first vibration frequency.

These coefficients are obtained for the nondimensional model. Consequently, in order to obtain a valid transfer function for the dimensional model, the following transformations must be made:

(76) $$\begin{array}{c} a^{*}\left(\lambda_{c}\right)=\frac{1}{T^{2}}a\left(\lambda_{c}\right);\;b^{*}\left(\lambda_{c}\right)=\frac{{\left(\rho L^{3}\right)}^{2}}{T^{6}}b\left(\lambda_{c}\right)\\ c^{*}\left(\lambda_{c}\right)=\rho^{2}L^{3}c\left(\lambda_{c}\right);\;d^{*}\left(\lambda_{c}\right)=\frac{\rho L^{3}}{T^{2}}d\left(\lambda_{c}\right) \end{array}$$

and the transfer function becomes

(77) $$G^{*}\left(s,\lambda_{c},\theta_{e}\right)=d^{*}\left(\lambda_{c}\right)+\frac{b^{*}\left(\lambda_{c}\right)+c^{*}\left(\lambda_{c}\right)g\sin\left(\theta_{e}\right)}{s^{2}+a^{*}\left(\lambda_{c}\right)}$$

Upon calculating Equation (77), a different representation of the transfer function is obtained:

(78) $$G_{a}\left(s,\lambda_{c},\theta_{e}\right)=K_{a}\left(\lambda_{c}\right)\cdot \frac{s^{2}+\beta^{2}\left(\lambda_{c},\theta_{e}\right)}{s^{2}+\alpha^{2}\left(\lambda_{c}\right)}$$

where $K_{a}\left(\lambda_{c}\right)$ is the gain of the model, $\beta (\lambda_{c},\theta_{e})$ represents the zeros, and $\alpha (\lambda_{c})$ denotes the poles, obtained from:

(79) $$K_{a}\left(\lambda_{c}\right)=d^{*}\left(\lambda_{c}\right)$$

(80) $$\beta \left(\lambda_{c},\theta_{e}\right)=\sqrt{a^{*}\left(\lambda_{c}\right)+\frac{b^{*}\left(\lambda_{c}\right)+c^{*}\left(\lambda_{c}\right)\cdot \hat{g}\cdot \sin\theta_{e}}{d^{*}\left(\lambda_{c}\right)}}$$

(81) $$\alpha \left(\lambda_{c}\right)=\sqrt{a^{*}\left(\lambda_{c}\right)}$$

**Figure 5:**
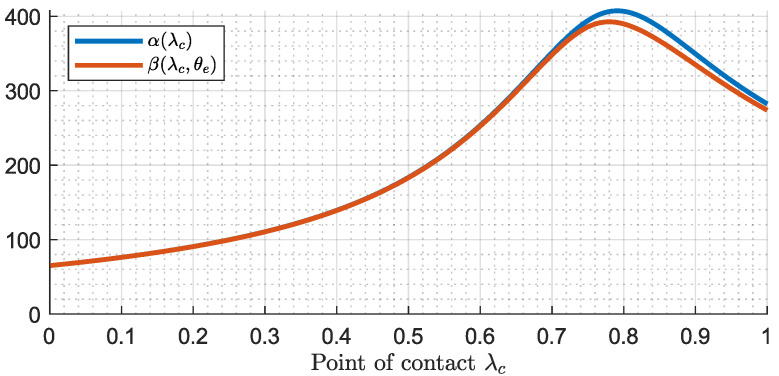
Values of $\alpha (\lambda_{c})$ and $\beta (\lambda_{c},\theta_{e})$ along $\lambda_{c}$.

Evaluating the values of $\alpha (\lambda_{c})$ and $\beta (\lambda_{c},\theta_{e})$ across the entire range $\lambda_{c}\in \left[0,\;1\right]$ and $\theta_{c}\in \left[-90^{\circ },\;90^{\circ }\right]$, it is demonstrated that $\alpha \left(\lambda_{c}\right)>\beta \left(\lambda_{c},\theta_{e}\right)$ consistently. This can be verified in Figure 5, which illustrates the evolution of $\alpha (\lambda_{c})$ and $\beta (\lambda_{c},\theta_{e})$ with respect to $\lambda_{c}$. This property is crucial for demonstrating the robustness of our force controller in the subsequent section.

**Figure A2:**
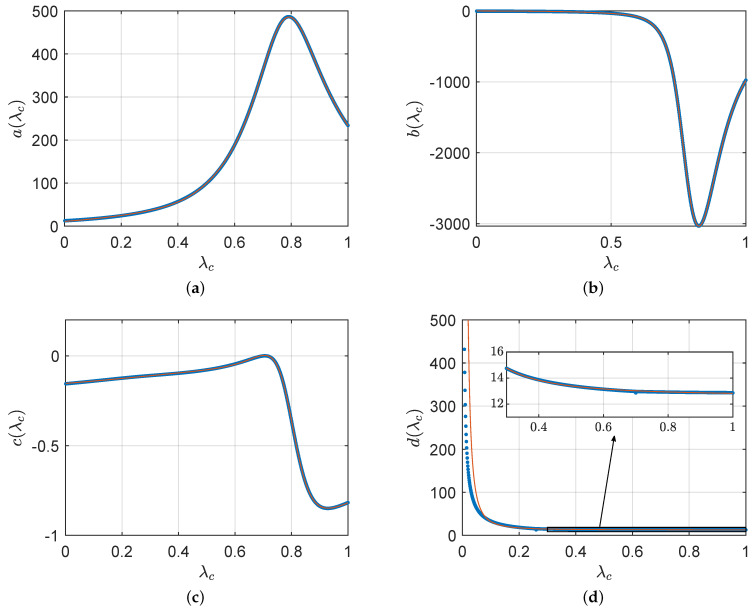
Coefficients of the transfer functions $a(\lambda_{c})$, $b(\lambda_{c})$, $c(\lambda_{c})$, and $d(\lambda_{c})$ with respect to the contact point $\lambda_{c}$: (**a**) $a(\lambda_{c})$, (**b**) $b(\lambda_{c})$, (**c**) $c(\lambda_{c})$ and (**d**) $d(\lambda_{c})$. Here, (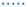) is the calculated value of the coefficients, whereas (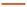) represents the adjusted functions.

Note that the value of $\beta (\lambda_{c},\theta_{e})$ barely changes with respect to $\theta_{e}$. This is because $c^{*}\left(\lambda_{c}\right)$ is significantly smaller than $b^{*}\left(\lambda_{c}\right)$, resulting in a $\beta \left(\lambda_{c},\theta_{e}\right)\approx \beta \left(\lambda_{c}\right)$. This observation can be checked in Figure A2 in Appendix C.

## 6. Control System

This section introduces a force control system for a single-link flexible robot operating under gravity. The objective is to regulate the force exerted by the flexible link on the environment, regardless of the contact point on the link. The control process is divided into three stages: (1) free motion control, where the link is servo-controlled until it makes contact with an object; (2) post-impact, where the link pushes against the object, gathering data from the force–torque sensor and using an estimator to identify where the contact point has been produced; and (3) force control, where the force exerted on the object is regulated using the information from the previous estimator. The system utilizes feedback from measurements of the motor’s position and force–torque at the base of the link.

Thus, the subsystems comprising the control system can be classified into two categories: controllers and estimators.

1. Controllers:
  (a) The motor position controller, which comprises the inner loop, regulates the dynamics of the motors between the motor angle $\theta_{m}\left(t\right)$ and its reference $\theta_{m}^{*}\left(t\right)$. This design ensures robustness against Coulomb friction, viscous friction, variations in link parameters, and external forces exerted on the link, allowing the system dynamics to be treated as a linear time-invariant system.
  (b) The force controller, comprising the outer loop, regulates the force applied by the antenna at the contact point to a desired value $F^{*}\left(t\right)$. This controller operates in conjunction with the inner loop. Thus, this outer loop uses feedback measurements of force–torque at the base of the link and command control signals that adjust the motor references $\theta_{m}^{*}\left(t\right)$.
2. Estimators:
  (a) The impact detector monitors the motor’s position and force–torque at the base of the link to detect the instant at which the antenna impacts an object.
  (b) The contact point estimator determines the point of the antenna at which contact has been detected.

The combination of these controllers and the estimators across the three stages of the control process is described.

In the first stage (free motion control), the inner loop is activated along with the impact detector, which continuously monitors the data. A programmed motor trajectory allows the antenna to perform a sweep. Then, the antenna moves freely until it makes contact, at which point the impact detector activates and triggers the transition to the second stage.

During the second stage (post-impact), a new reference is set for the inner loop, causing the motors to increase their position relative to the angle at which the contact has been detected, thereby ensuring that the antenna continues to exert pressure on the object. Then, the system remains steady for a predetermined period of time, collecting force–torque data from the base of the link until the contact point estimator identifies the point at which the antenna is pushing against the object.

Following contact point estimation, the third stage (force control) commences. The force control strategy incorporates two nested control loops: the inner loop and the outer loop. The inner loop regulates the motor position, while the outer loop indirectly controls the exerted force by adjusting the torque at the base of the link. Once the distance from the contact point to the joint is estimated, the outer loop sets the desired torque at the base of the link as a reference. This reference is calculated by multiplying the estimated distance by the desired force.

Detailed descriptions of all subsystems involved in the control process are provided below.

### 6.1. Motor Control Inner Loop

The inner loop is designed to control the position angle of the actuators so that the dynamics between the motor position and its reference become an approximately linear time-invariant system. This control is insensitive to gravity and external forces acting on the antenna and remains active throughout the entire control process. This control system has been utilized in both free- and constrained-motion scenarios (e.g., [39]), demonstrating its robustness and effectiveness. The structure incorporates PID controllers with a low-pass filter term, ensuring excellent trajectory tracking, compensating for disturbances such as unmodeled friction components, and maintaining robustness against parameter uncertainties. This provides precise and rapid motor positioning responses. Additionally, it includes a compensator for the nonlinear friction of the motor (stiction) to avoid motor dead-zones and a compensator for the estimated coupling torque caused by the force exerted by the link on the contacted object.

An algebraic design methodology allows for the arbitrary placement of the four poles and two zeros of the closed-loop system. By aligning the zeros and poles at the same position, denoted as $p_{m}$, and assuming that the compensators perfectly cancel the nonlinearities, the inner closed-loop dynamics are defined as follows:

(82) $$G_{M}\left(s\right)=\frac{\theta_{m}\left(s\right)}{\theta_{m}^{*}\left(s\right)}=\frac{1}{{\left(1+\varepsilon \cdot s\right)}^{2}};\;\varepsilon ={\left(-p_{m}\right)}^{-1};$$

This configuration allows for very rapid motor movements if the absolute values of the poles $p_{m}$ are set high, provided that actuator saturation is avoided.

### 6.2. Impact Detector

Determining the precise moment of contact is crucial for initiating the mechanisms that estimate the contact point on the beam. In robotics, several mechanisms have been proposed that detect a collision by monitoring measured variables that exceed a threshold: [40] for rigid-link robots and [41] for flexible-link robots. Contact instants have also been estimated in artificial antennae designed to mimic insect behavior [42]. These experiments employed a two-axis acceleration sensor positioned at the antenna’s tip to measure link vibrations and gather information about contact. Information regarding contact was derived from analyzing vibration frequencies. In the case of our flexible antenna, we utilized a mechanism that predicts real-time coupling torque during the antenna’s free movement. This mechanism involves comparing the predicted torque with sensor measurements in real time. The equation used to estimate coupling torque is derived from the dynamics of the flexible antenna link. A brief outline of this estimator is given below.

Consider the measured coupling torque $\vec{\Gamma^{m}}\left(t\right)={\left(\Gamma_{x}^{m}\left(t\right),\Gamma_{y}^{m}\left(t\right),\Gamma_{z}^{m}\left(t\right)\right)}^{⊺}$ provided by the F-T sensor at the base of the link. Denote the effect of gravity on the beam provided in the F-T sensor frame as $\vec{\Gamma^{g}}\left(t\right)={\left(0,0.5\cdot \rho \cdot L\cdot g\cdot \cos\left(\theta_{m_{2}}\left(t\right)\right),0\right)}^{⊺}$, with the product ρ·L being the mass of the antenna and $\theta_{m_{2}}\left(t\right)$ the elevation motor angle. Define $\vec{\Gamma^{e}}\left(t\right)$ as a real-time estimation of the coupling torque during free-movement mode, assuming no gravity, obtained from Equations (31) and (32). The residual error between the measured and the estimated coupling torques can be defined as:

(83) $$r_{\Gamma }\left(t\right)=\vec{\Gamma^{m}}\left(t\right)-\left(\vec{\Gamma^{e}}\left(t\right)+\vec{\Gamma^{g}}\left(t\right)\right)$$

Then, contact is produced at the instant $t_{i}$ at which the absolute value of the time derivative of the magnitude of the residual error exceeds the threshold:

(84) $$\left|\frac{dr_{\Gamma }\left(t\right)}{dt}\right|>r_{\Gamma }^{max}$$

where the threshold $r_{\Gamma }^{max}$ is determined experimentally.

### 6.3. Contact Point Estimation

We propose determining the contact point of the antenna, where it makes contact with the surface of an object, using the algorithm outlined in [39]. This algorithm combines two estimators. The first one relies on the relation between the lowest natural frequency of the oscillations experienced by the antenna after an impact, $\omega_{1}$, and the contact position, $l_{c}$, as described in Section 4 and represented in Figure 4. This relationship is usually tabulated, allowing for a quick estimation of the contact point from the frequency value. Although this method gives a very precise estimation, it can sometimes yield two possible solutions. To resolve this ambiguity, a second estimator is employed. This one estimates the contact point using the static force and torque measurements of the sensor and the relation between these magnitudes. Since torque is the product of force and distance, it straightforwardly determines the application point. While this method may be less precise than the first, it effectively distinguishes between the two potential solutions provided by the initial estimator.

The contact point estimation process begins when the impact detector triggers the transition from the first stage (free motion of the antenna) to the second stage (post-impact). The antenna pushes against the object and remains steady for a determined period of time Δt, during which the F-T sensor registers the oscillations of the antenna. Subsequently, Fast Fourier Transform (FFT) is performed on the data to determine the first vibration frequency $\omega_{1}$, and the contact point $l_{c}$ is obtained from the tabulated data, as depicted in Figure 4. In cases where two potential contact points are identified, the second estimator computes the contact point, determining which of the initial estimates is correct.

The precision of obtaining the frequency $\omega_{1}$ by performing FFT on the registered data is inversely proportional to the length of the data vector. Longer data vectors provide greater precision, but also increase algorithm execution time. Therefore, a balance must be struck in defining the data registration time, Δt.

### 6.4. Force Control Outer Loop

Force control is indirectly achieved by controlling the torque at the base of the antenna. If the force we aim to exert is $F^{*}\left(t\right)$ at contact point $l_{c}$, then a moment at the base of the antenna $\Gamma^{*}\left(t\right)=F^{*}\left(t\right)\cdot l_{c}$ should be exerted. The feedback measure from the F-T sensor Γ(t) and the rest of the measures are modified to correct the gravity effect of the antenna system, as is performed for the impact detector in Section 6.2. Thus, the feedback signal is obtained from

(85) $$\vec{\Gamma }\left(t\right)=\vec{\Gamma^{m}}\left(t\right)-\vec{\Gamma^{g}}\left(t\right),\;\vec{\Gamma^{g}}\left(t\right)={\left(0,0.5\cdot \rho \cdot L\cdot g\cdot \cos\left(\theta_{m_{2}}\left(t\right)\right),0\right)}^{⊺}$$

(86) $$\vec{F}\left(t\right)=\vec{F^{m}}\left(t\right)-\vec{F^{g}}\left(t\right),\;\vec{F^{g}}\left(t\right)={\left(-\rho \cdot L\cdot g\cdot \sin\left(\theta_{m_{2}}\left(t\right)\right),0,-\rho \cdot L\cdot g\cdot \cos\left(\theta_{m_{2}}\left(t\right)\right)\right)}^{⊺}$$

with $\vec{\Gamma^{m}}\left(t\right)={\left(\Gamma_{x}^{m}\left(t\right),\Gamma_{y}^{m}\left(t\right),\Gamma_{z}^{m}\left(t\right)\right)}^{⊺}$, $\vec{F^{m}}\left(t\right)={\left(F_{x}^{m}\left(t\right),F_{y}^{m}\left(t\right),F_{z}^{m}\left(t\right)\right)}^{⊺}$ being the measured coupling torques and forces, respectively, provided by the F-T sensor at the base of the link, and $\vec{\Gamma^{g}}\left(t\right)$, $\vec{F^{g}}\left(t\right)$ the torque and force effects of gravity on the beam provided in the F-T sensor frame, where the product ρ·L is the mass of the antenna and $\theta_{m_{2}}\left(t\right)$ the elevation motor angle.

**Figure 6:**
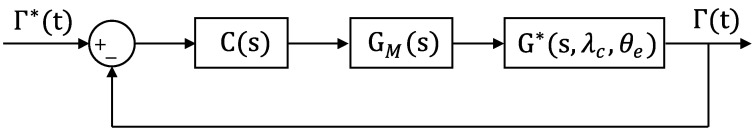
Force control outer loop scheme.

Figure 6 presents the outer loop of the system, which is composed of the following:

1. The transfer function $G^{*}\left(s,\lambda_{c},\theta_{e}\right)$ from Equation (77) describing the dynamics of the antenna in contact with an object.
2. The motor control inner loop $G_{M}\left(s\right)$ whose dynamics are described by Equation (82).
3. A controller C(s) whose robustness would be justified if it were of the PI type:
  (87) $$C\left(s\right)=\frac{K_{c}\cdot \left(s+a_{c}\right)}{s}\;,\;K_{c},a_{c}>0$$
  which verifies the robustness condition
  (88) $$0<a_{c}\cdot \varepsilon <2$$

This control system robustly stabilizes the dynamics of the robot under contact, maintaining stability for any pair of values where $0<\lambda_{c}\leq 1$ and $-90^{\circ }\leq \theta_{c}\leq 90^{\circ }$, as well as for any uncertainties in the mechanical parameters of the antenna. The next subsection will prove the above robustness conditions.

#### 6.4.1. Stability Robustness Condition of C(s)

The robust stability of the PI force controller (87) is designed using the Routh–Hurwitz criterion, e.g., [43].

The closed-loop characteristic equation is

(89) $$1+K_{a}\left(\lambda_{c}\right)\cdot \frac{s^{2}+\beta^{2}\left(\lambda_{c},\theta_{e}\right)}{s^{2}+\alpha^{2}\left(\lambda_{c}\right)}\cdot \frac{K_{c}\cdot \left(s+a_{c}\right)}{s}\cdot \frac{1}{{\left(1+\varepsilon \cdot s\right)}^{2}}=0$$

We hereafter omit the arguments of $K_{a}$, β, and α for the sake of simplicity. Then, the characteristic polynomial is

(90) $$\begin{array}{c} \varepsilon^{2}\cdot s^{5}+2\cdot \varepsilon \cdot s^{4}+\left(1+\alpha^{2}\cdot \varepsilon^{2}+K_{a}\cdot K_{c}\right)\cdot s^{3}+\left(2\cdot \varepsilon \cdot \alpha^{2}+K_{a}\cdot K_{c}\cdot a_{c}\right)\cdot s^{2}+\\ \left(\alpha^{2}+K_{a}\cdot K_{c}\cdot \beta^{2}\right)\cdot s+K_{a}\cdot K_{c}\cdot a_{c}\cdot \beta^{2}=0 \end{array}$$

In order to assess the closed-loop stability, we must first check that all the coefficients of this polynomial are positive. This is easily verified, since α, β, $K_{a}$, $K_{c}$, $a_{c}$, and ε are positive. Next, we calculate the Routh table, giving the following terms in the first column:

- Term $s^{5}$ gives $\varepsilon^{2}>0$.
- Term $s^{4}$ gives 2·ε>0.
- Term $s^{3}$ gives $2+K_{a}\cdot K_{c}\cdot \left(2-a_{c}\cdot \varepsilon \right)$. It is easy to see that $2-a_{c}\cdot \varepsilon >0$ is a sufficient condition to make this term positive.
- Term $s^{2}$ gives $2\cdot K_{a}\cdot K_{c}\cdot a_{c}+K_{a}^{2}\cdot K_{c}^{2}\cdot a_{c}\cdot \left(2-a_{c}\cdot \varepsilon \right)+2\cdot K_{a}\cdot K_{c}\cdot \varepsilon \cdot \left(\alpha^{2}-\beta^{2}\right)\cdot \left(2-a_{c}\cdot \varepsilon \right)$. It is easy to see that $2-a_{c}\cdot \varepsilon >0$ with α>β are sufficient conditions to make this term positive.
- Term $s^{1}$ gives $\Psi =\psi_{1}\cdot \psi_{2}-\psi_{3}\cdot \psi_{4}$, where
  (91) $$\begin{array}{c} \psi_{1}=2\cdot K_{a}\cdot K_{c}\cdot a_{c}+K_{a}^{2}\cdot K_{c}^{2}\cdot a_{c}\cdot \left(2-a_{c}\cdot \varepsilon \right)+2\cdot K_{a}\cdot K_{c}\cdot \varepsilon \cdot \left(\alpha^{2}-\beta^{2}\right)\cdot \left(2-a_{c}\cdot \varepsilon \right)\\ \psi_{2}=2\cdot \alpha^{2}+K_{a}\cdot K_{c}\cdot \beta^{2}\cdot \left(2-a_{c}\cdot \varepsilon \right)\\ \psi_{3}=2+K_{a}\cdot K_{c}\cdot \left(2-a_{c}\cdot \varepsilon \right)\\ \psi_{4}=K_{a}\cdot K_{c}\cdot a_{c}\cdot \beta^{2}\cdot \left(2+K_{a}\cdot K_{c}\cdot \left(2-a_{c}\cdot \varepsilon \right)\right) \end{array}$$
  Note that if $2-a_{c}\cdot \varepsilon >0$ and α>β are verified, then $\psi_{i}$, 1≤i≤4 is positive. Moreover, $\psi_{2}/\beta^{2}-\psi_{3}=2\cdot \frac{\alpha^{2}-\beta^{2}}{\beta^{2}}>0$ and $\psi_{1}\cdot \beta^{2}-\psi_{4}=2\cdot K_{a}\cdot K_{c}\cdot \beta^{2}\cdot \varepsilon \cdot \left(\alpha^{2}-\beta^{2}\right)\cdot \left(2-a_{c}\cdot \varepsilon \right)>0$. Taking into account that $\Psi =\psi_{1}\cdot \beta^{2}\cdot \frac{\psi_{2}}{\beta^{2}}-\psi_{3}\cdot \psi_{4}$, this implies that Ψ>0.
- Term $s^{0}$ gives $\varepsilon \cdot K_{a}\cdot K_{c}a_{c}\cdot \beta^{2}\cdot \left(2+K_{a}\cdot K_{c}\cdot \left(2-a_{c}\cdot \varepsilon \right)\right)$. And, again, it is easy to see that $2-a_{c}\cdot \varepsilon >0$ is a sufficient condition to make this term positive.

Therefore, considering that the property α>β seen in the previous section is verified, we have proven that a PI controller provides force control with robust stability if the following condition

(92) $$0<a_{c}\cdot \varepsilon <2$$

is verified.

#### 6.4.2. Design Methodology of C(s)

In this particular application, an algebraic methodology is followed to adjust the parameters $K_{c}$, $a_{c}$ of the controller C(s) (87). The characteristic equation of the system is

(93) $$Q\left(s,\lambda_{c},\theta_{e}\right)=1+C\left(s\right)\cdot G_{M}\left(s\right)\cdot G_{a}\left(s,\lambda_{c},\theta_{e}\right)=0$$

Calculating Equations (93) with (87), (82) and (78), while omitting $\lambda_{c}$, $\theta_{e}$ for clarity, yields:

(94) $$Q\left(s\right)=s\cdot {\left(1+\varepsilon \cdot s\right)}^{2}\cdot \left(s^{2}+\alpha^{2}\right)+K_{c}\cdot \left(s+a_{c}\right)\cdot K_{a}\cdot \left(s^{2}+\beta^{2}\right)=0$$

The two parameters $K_{c}$ and $a_{c}$ of the controller C(s) need to be adjusted. Thus, if a double pole of the system is selected and placed at $p_{F}$, the following relations need to be accomplished:

(95) $$Q(p_{F})=0$$

(96) $${\left.\frac{dQ(s)}{ds}\right|}_{s=p_{F}}=0$$

From Equation (95), the parameter $K_{c}$ based on $a_{c}$ is obtained:

(97) $$K_{c}=\frac{-p_{F}\cdot {\left(1+\varepsilon \cdot p_{F}\right)}^{2}\cdot \left(p_{F}^{2}+\alpha^{2}\right)}{K_{a}\cdot \left(p_{F}^{2}+\beta^{2}\right)\cdot \left(p_{F}+a_{c}\right)}$$

Finally, by calculating Equation (96) and including (97), the parameter $a_{c}$ is obtained:

(98) $$a_{c}={\left(\frac{3\cdot p_{F}^{2}+\alpha^{2}}{p_{F}^{2}+\alpha^{2}}+\frac{2\cdot p_{F}\cdot \varepsilon }{1+\varepsilon \cdot p_{F}}-\frac{2\cdot p_{F}^{2}}{p_{F}^{2}+\beta^{2}}\right)}^{-1}-1$$

#### 6.4.3. Justification on Tuning the Outer Loop Considering the above Robust Stability Condition

Expressions (97) and (98) enable real-time tuning of the PI controller (87) once $\lambda_{c}$ and $\theta_{e}$ have been estimated. We recall that $\theta_{e}$ has minimal influence on the parameters of transfer functions $G_{a}\left(s,\lambda_{c},\theta_{e}\right)$. However, if necessary, it can be determined by combining measurements from the motor encoders and an inclinometer mounted on the base of the haptic system. The tuning process aims to achieve the same closed-loop poles independently of the contact point $\lambda_{c}$. Nevertheless, this ideal situation cannot be fully achieved in practice due to the following factors:

1. Variations in the estimation of the frequency $\omega_{1}$ from measured data can lead to incorrect contact point estimations. As previously mentioned, the precision of the FFT depends on the length of the data vector, defined by Δt. Since this period cannot be set too high, it inevitably introduces imprecision in contact point estimation.
2. Modelling errors cause variations in the curve relating the contact point and first vibration frequency of the antenna, represented in Figure 4.
3. The transfer function $G_{a}\left(s,\lambda_{c},\theta_{e}\right)$ (77) obtained from the model is a simplification of the full system, truncated to the first mode of vibration. This simplification introduces modeling errors in the parameters of this transfer function, leading to non-optimal calculations of controller parameters.

These issues can result in unstable outer loop control systems if a robustness condition is not imposed in the design of C(s). An unstable outer loop can cause undesirable and dangerous behavior, potentially exerting excessive force and risking the integrity of the robot. We have proven that condition (92) guarantees closed-loop stability in all these cases. Moreover, it ensures limited deterioration of the transient response in cases of mismatch.

## 7. Robot Parameters and Experimental Results

**Figure 7:**
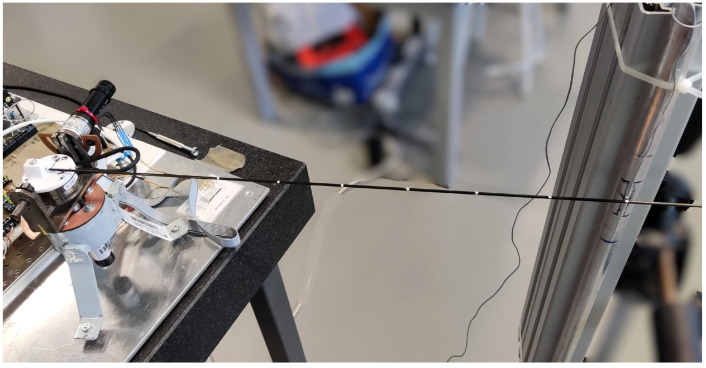
Experimental setup. In this photo, the system is performing an azimuthal (horizontal) movement with the sensing antenna hitting the steel cylinder at $\lambda_{c}=0.9$ of the antenna.

Experiments are conducted to test various contact points and programmed reference forces. Both degrees of freedom of the robot (azimuthal and elevation movements) are evaluated. Specifically, a set of seven different contact points ranging from $\lambda_{c}=0.3$ to $\lambda_{c}=0.9$ and three levels of reference forces $|F^{*}|=\left(0.05,0.10,0.15\right)$ N are tested between one and five times for each degree of freedom. An image of the experimental setup is shown in Figure 7, where the system is performing an azimuthal (horizontal) movement with the sensing antenna making contact with a steel cylinder at $\lambda_{c}=0.9$ of the antenna.

In this section, we first detail the main parameters of the system and the control process. Then, we present the experimental results obtained from the different algorithms at each stage of the control process.

### 7.1. Parameters of the System

Table 1 shows the parameters of the two motors of the system, where *Motor 1* and *Motor 2* refer to the azimuthal and elevation motors, respectively.

Table 2 details the characteristics of the antenna. Note that the link flexural rigidity EI, as defined previously, is a product of Young’s Modulus *E* and the area moment of inertia *I*.

Finally, Table 3 shows the most important parameters of each control system:

- First, in the motor control inner loop, the closed-loop system’s poles $p_{m}$ are placed at the same value for both the azimuthal and elevation motors to achieve homogeneous behavior of the system in both degrees of freedom.
- Second, the impact detector threshold $r_{\Gamma }^{max}$, which has power units (N·m/s), is determined experimentally based on the maximum value of (84) obtained in the free-motion experiments, with an added security margin.
- Third, in the contact point estimator, a time of Δt=0.7 s is chosen as it provides sufficient FFT precision while allowing the algorithm to execute quickly enough. In this case, the relation between $\omega_{1}$ and $l_{c}$, described in Section 4 and represented in Figure 4, is tabulated to allow for quick estimation of the contact point from the frequency value.
- And fourth, in the force control outer loop, the closed-loop system’s poles $p_{F}$ are placed to achieve the fastest outer loop response possible while satisfying the robustness condition (92). Furthermore, the different values of the parameters $K_{c}\left(\lambda_{c},\theta_{e}\right)$ (97) and $a_{c}\left(\lambda_{c},\theta_{e}\right)$ (98) of the force controller C(s) (87) are tabulated to facilitate quick tuning of the outer loop during experimentation.

### 7.2. Results

**Figure 8:**
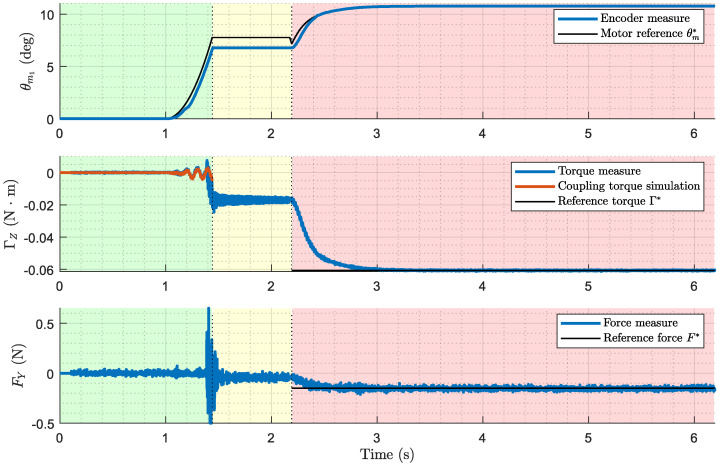
Complete experimental results. Motor angular position (inner loop), measured torque and force (outer loop) along the three stages of the control process. Case: azimuthal displacement, contact point $\lambda_{c}=0.9$, programmed force of $F^{*}=0.15$ N.

The results of the experiment depicted in the photo in Figure 7, where the antenna performs azimuthal displacement, contacts the cylinder at $\lambda_{c}=0.9$, and pushes with a programmed force of $F^{*}=0.15$ N, are represented in Figure 8. The data illustrate the complete control process, from the first stage of free motion control, through post-impact data acquisition in the second stage, to the force control in the third stage. Hereafter, the graphical results presented below belong to this same experiment.

#### 7.2.1. First Stage Results: Impact Detector

**Figure 9:**
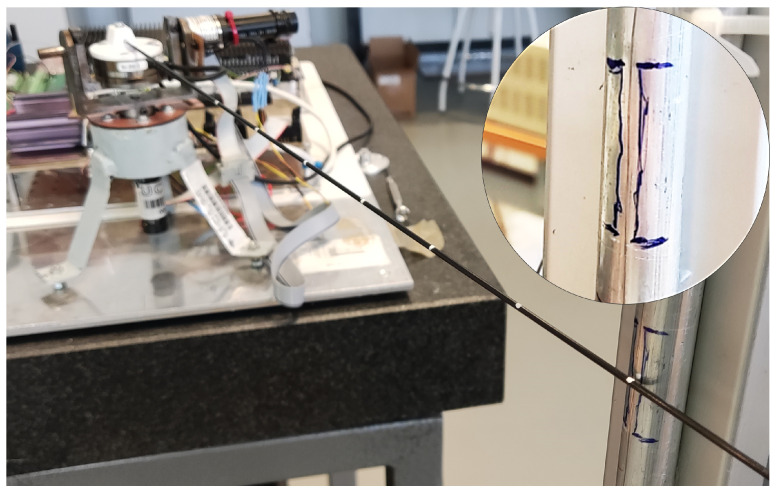
Analog impact detector.

The time required for the impact detector to detect contact is measured using a special setup involving the object that the antenna impacts. This setup consist of a thin copper wire attached very close to the surface of the steel cylinder, but not touching it. The cylinder is wired to the digital input of the DAQ system and is set to zero volts. The wire is connected to an output port of the DAQ supplying 5 volts. When the antenna hits the cylinder, it also pushes the wire towards the cylinder surface, causing an electrical connection between the wire and the cylinder. This results in a voltage change in the digital input of the DAQ system connected to the cylinder, registering the exact instant $t_{i_{A}}$ at which the antenna contacts the cylinder. This setup is hereafter referred to as the analog impact detector, and a detailed image of it is shown in Figure 9.

**Figure 10:**
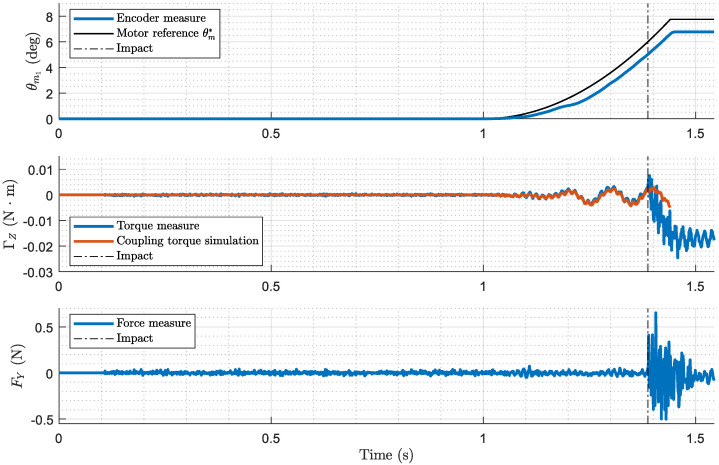
First stage experimental results (free-motion control). Motor angular position (inner loop), measured torque and force (outer loop). Case: azimuthal displacement, contact point $\lambda_{c}=0.9$, programmed force of $F^{*}=0.15$ N.

Figure 10 shows the performance of the system during the first stage, where the motors control the movement of the antenna, seeking the space until it makes contact with the cylinder. The figure includes plots of the motor reference versus encoder signal, measured torque versus torque simulated by the detector, and measured force applied to the cylinder. It also represents the moment at which the analog impact detector ($t_{i_{A}}$) detects contact.

**Figure 11:**
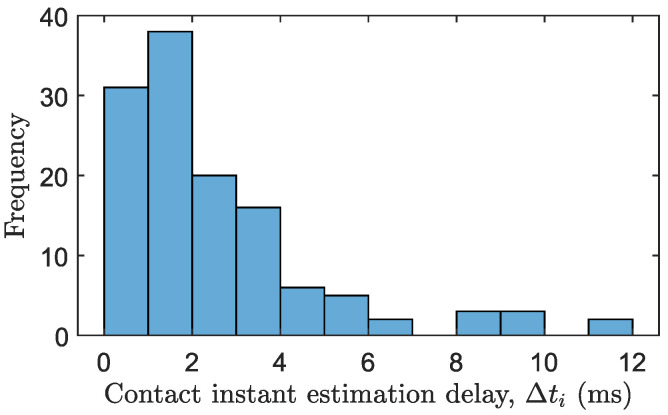
Histogram of the delay in estimating the contact instant $\Delta t_{i}$ for all experiments.

Table 4 summarizes the mean results and the standard deviation of all experiments regarding the delay in estimating the contact instant. The time required for the impact detector to detect contact is calculated as $\Delta t_{i}=t_{i}-t_{i_{A}}$, where $t_{i}>t_{i_{A}}$. Alongside this table, a histogram of $\Delta t_{i}$ for all experiments is shown in Figure 11. The histogram illustrates that the most frequent time estimation delay falls between 0 and 2 milliseconds.

#### 7.2.2. Second Stage Results: Contact Point Estimation

**Figure 12:**
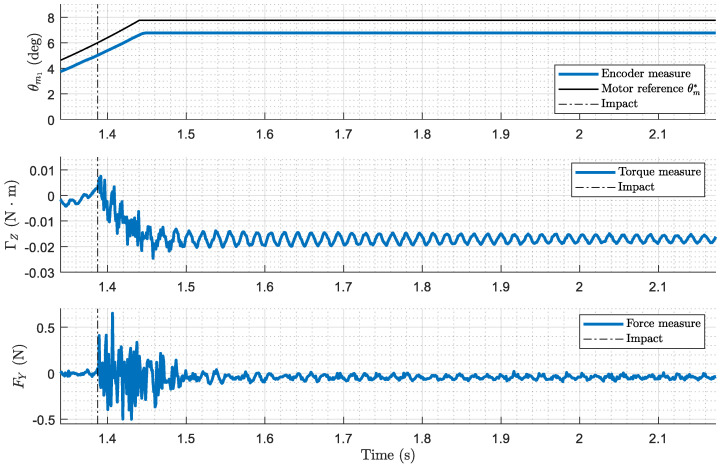
Second stage experimental results (post-impact, data acquisition). Motor angular position (inner loop), measured torque and force (outer loop). Case: azimuthal displacement, contact point $\lambda_{c}=0.9$, programmed force of $F^{*}=0.15$ N.

The experimental setup is positioned in each experiment for the impact to occur at a programmed position of the antenna. Specifically, a set of seven different contact points from $\lambda_{c}=0.3$ to $\lambda_{c}=0.9$ are measured and marked on the antenna with a white point (see Figure 7). Figure 12 shows the collected data during this second stage, where the antenna remains steady, pushing the cylinder for a determined period of time Δt. This parameter determines the precision with which the FFT determines the frequency. The precision in the FFT frequency is calculated as the maximum frequency read $f_{max}$, which is half of the frequency of the system, divided by the length of the registered data $L_{data}$, related to Δt such that

(99) $$\Delta f=\frac{f_{max}}{L_{data}}=\frac{\frac{1}{2}\cdot \frac{1}{T_{s}}}{\frac{1}{2}\cdot \frac{\Delta t}{T_{s}}}=\frac{1}{\Delta t}$$

Taking into account the relation between $\omega_{1}$ and $l_{c}$ described in Section 4 and represented in Figure 4, the maximum Δf that can be selected varies between 1.4 and 1.5 Hz. This data are obtained considering less than a 2% error in the length of the antenna when estimating the contact point, which corresponds to approximately 10 mm. Thus, the data acquisition time selected for the second stage is Δt=0.7 s.

**Figure 13:**
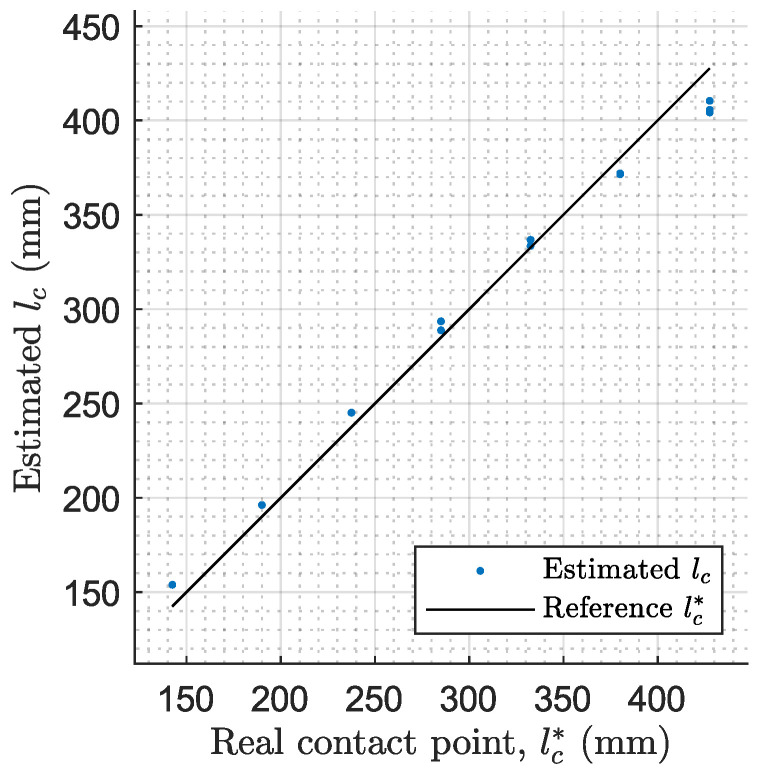
Comparison between real contact point reference $l_{c}^{*}$ and the estimated contact points $l_{c}$ for all experiments.

Table 5 summarizes the mean values of the estimated contact point and its errors for all the experiments. It can be observed that the mean absolute errors do not exceed the limit of 10 mm, which is approximately 2%. Alongside the table, Figure 13 shows the estimated contact points $l_{c}$ for all experiments in comparison with the real contact point reference $l_{c}^{*}=\lambda_{c}\cdot L$. It can be seen that the estimator provides accurate results.

#### 7.2.3. Third Stage Results: Performance of Force Control

**Figure 14:**
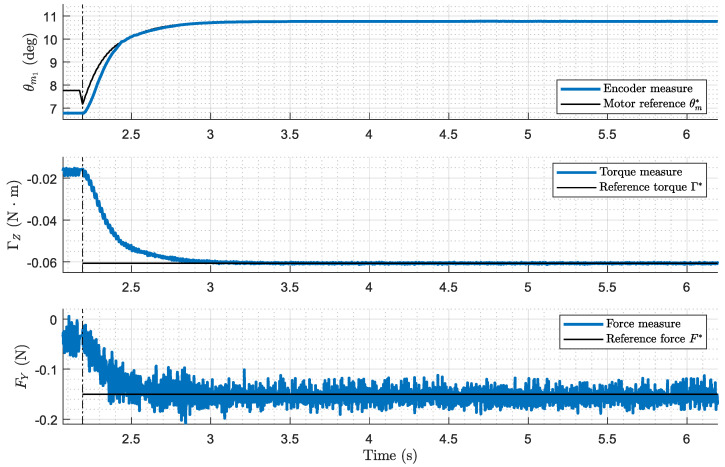
Third stage experimental results (force control). Motor angular position (inner loop), measured torque and force (outer loop) Case: azimuthal displacement, contact point $\lambda_{c}=0.9$, programmed force of $F^{*}=0.15$ N.

Figure 14 illustrates the performance of the control system during this third stage, where the antenna applies a specified force to the cylinder. During this phase, both the inner and outer loops of the control system operate concurrently. The programmed force is the reference of the outer loop, and the control signal that it generates results in the input reference of the inner loop. Figure 14 demonstrates that the system operates effectively with zero steady-state errors.

As explained earlier, the outer loop controls the torque at the base of the antenna with the reference $\Gamma^{*}\left(t\right)=F^{*}\left(t\right)\cdot l_{c}$, where $F^{*}\left(t\right)$ is the desired force and $l_{c}$ is the estimated contact point from the previous stage. A PI controller is used, which achieves zero steady-state error in torque for every experiment conducted. However, variations in the estimation of the contact point $l_{c}$ lead to two issues: (1) tuning C(s) with non-optimal parameters, and (2) setting an incorrect reference torque $\Gamma^{*}\left(t\right)$.

**Figure 15:**
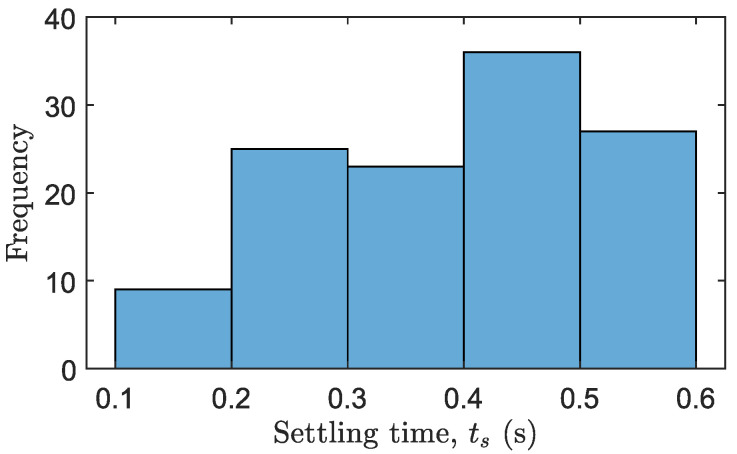
Histogram of the settling time responses $t_{s}$ of the outer loop.

The first issue affects the transient response of the resultant system, as it does not operate as quickly as theoretically predicted. Ideally, the settling time $t_{s}$ of the outer loop response, obtained from simulations, is $t_{s}=0.135$ s. This ideal result can be compared with the data in Table 6, which presents the mean settling time measured in each experiment. Additionally, Figure 15 shows a histogram of the settling times $t_{s}$ obtained for all experiments.

Finally, the second issue affects the steady-state response of the system. Since the estimation $l_{c}$ of the contact point may introduce errors compared with the real contact point $l_{c}^{*}$ (see Figure 13), setting the reference torque $\Gamma^{*}\left(t\right)$ causes the control to push the cylinder with a force of $F\left(t\right)=F^{*}\left(t\right)\cdot \left(l_{c}/l_{c}^{*}\right)$, which does not exactly correspond to the desired applied force $|F^{*}|=\left(0.05,0.10,0.15\right)$ N. The percentage error between the desired force $F^{*}$ and the real applied force *F* is calculated as:

(100) $$e_{F}\left(\%\right)=100\cdot \left|\frac{F^{*}-F}{F^{*}}\right|=100\cdot \left|1-\frac{l_{c}}{l_{c}^{*}}\right|$$

**Figure 16:**
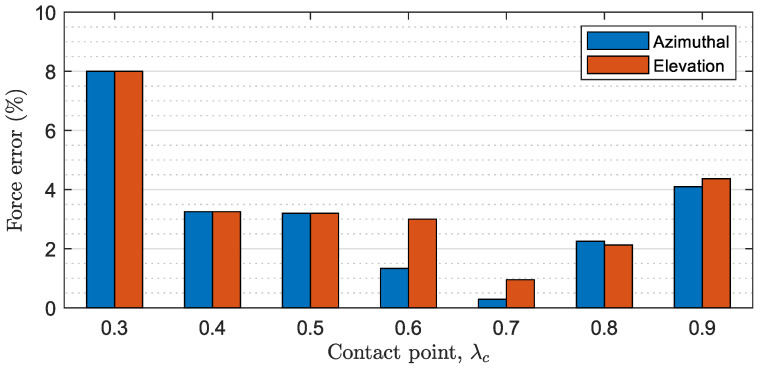
Summary of stage 3 results (II): mean percentage force error obtained for azimuthal and elevation experiments.

The mean percentage error of force is calculated considering the errors introduced by the estimation of the contact point in all the experiments conducted, and it is illustrated in Figure 16. These results are consistent with the errors observed experimentally (e.g., in Figure 14).

## 8. Discussion

This paper developed and tested a precise force control mechanism for a haptic device comprising a flexible link that rotates around one of its ends, resembling the antennae found in many insects. The link, with a distributed mass, executes azimuthal and elevation movements influenced by gravity. Contact with an object can occur at any intermediate point along the link. It is crucial in this context to regulate the force exerted by the antenna on the object to facilitate tasks such as object identification or moving an object. Previous works have addressed force control only when the contact is at the tip. Our work makes several significant contributions to the state of the art because, for the first time, (1) precise force control at intermediate points of a link is achieved; (2) a condition to design robustly stable controllers is obtained, i.e., controllers that maintain acceptable performance independently of the features of the controlled dynamics, that highly change with the contact point at the link; (3) we prove that simple PI controllers verifying this condition achieve such robustness stability; and (4) this control system yields satisfactory experimental results. Moreover, a lumped-mass model (with more than a lumped mass) of a flexible link in the context of contact with an object at an intermediate point is developed for the first time. This model is general because it is developed for a normalized beam.

Next, we specify the roles played by the dynamic models developed in this work. In the first scenario, in which the antenna moves freely, vibrating without suffering any contact, the obtained free model is used to predict (by simulating this model) the coupling torque. This prediction serves to compute the residue used by the impact detector for estimating the impact instant. In the second scenario, the antenna presses the object in a motor control fashion, without employing a flexible-link model. In the third scenario, the parameters of the PI controller are tuned using the family of approximate models obtained for the case of contact (these contact models are different in terms of the function of $\lambda_{c}$, and the forms of their dependence on $\lambda_{c}$ vary in terms of the function of the interval between masses that is being considered or whether $\lambda_{c}$ corresponds to the position of one of the masses of the lumped dynamics model). Moreover, we mention that the obtained contact models played decisive roles in obtaining the family of robust controllers: (1) these models yielded truncated models with two imaginary poles and two imaginary zeros that were used in the closed-loop stability assessment, and (2) they allowed us to establish a property whereby the zeros are closer to the origin of the S-complex plane than the poles, a property that was crucial for obtaining the robustness condition.

We highlight that we have designed a broader control system in which the PI force controller is embedded. It includes also an impact detector and a real-time estimator of the contact point. The experiments conducted using this whole system demonstrate the effectiveness of this methodology, ensuring the stability of the system and achieving minimal force error at the contact point. Figure 14 shows a mean error in the steady state of nearly null as consequence of using a PI controller. However, since the values of the exerted forces are low, noticeable noise can be observed in the figure because these values are not far away from the accuracy level of the force–torque sensor.

Next, we mention some limitations of the system. The first one is the precision of the controlled force, which depends on the strain gauge offset of the F-T sensor and, as previously mentioned, on the inaccuracy produced in the estimation of the contact position, which introduces a small error in the calculated torque reference. Another limitation is the assumption of small deflection. If this assumption were violated, the model obtained in Section 3 would be incorrect and the dynamics would become nonlinear. Finally, a third limitation is the assumption of the constant cross-section of the antenna. Other behaviors can be obtained using conical antennae. In this case, Section 3 should be developed assuming a decreasing cross section radius.

Finally, we mention that potential applications of the proposed force control exceed the haptic antennae case. This can be applied in other robotic scenarios like the following: (1) in biomimetics, where it can be used to design robotic birds that grasp objects with their beaks; (2) in industrial robots, where it can be used to design hands with flexible fingers that grasp objects with a programmed force, with contact at intermediate points of the fingers; and (3) in robot-assisted surgery, where a required force has to be exerted when the robot contacts an organ.

## Figures and Tables

**Table 1:** Parameters of the motors.

Parameter	Symbol	Value Motor 1	Value Motor 2	Units
Electromechanical constant	$K_{m}$	0.003	0.003	N·m/V
Rotational inertia	$J_{0}$	$6.18\times 10^{-7}$	$1.85\times 10^{-7}$	kg·m
Viscous friction	*v*	$3.04\times 10^{-6}$	$2.85\times 10^{-6}$	N·m·s
Dead-zone	$V_{f}$	0.40	0.45	V
Saturation	$v_{sat}$	1.20	1.20	V

**Table 2:** Parameters of the antenna.

Parameter	Symbol	Value	Units
Length	*L*	475	mm
Diameter	⌀	1.98	mm
Young’s Modulus	*E*	$1.15\times 10^{11}$	N/m^2^
Area moment of inertia	*I*	$7.54\times 10^{-13}$	m^4^
Linear density	ρ	$5\times 10^{-3}$	kg/m

**Table 3:** Parameters of the control system.

Control System	Parameter	Symbol	Value
Inner loop	Closed-loop system’s poles	$p_{m}$	−60
Impact detector	Estimator threshold (N·m/s)	$r_{\Gamma }^{max}$	5
Contact point estimator	Data registration time (s)	Δt	0.7
Outer loop	Closed-loop system’s poles	$p_{F}$	−40

**Table 4:** Summary of stage 1 results: mean and standard deviation σ of all experiments regarding the delay in estimating the contact instant.

DOF	Azimuthal		Elevation	
$\lambda_{c}$	$\bar{\Delta t_{i}}$ (ms)	$\sigma t_{i}$ (ms)	$\bar{\Delta t_{i}}$ (ms)	$\sigma t_{i}$ (ms)
**0.3**	1.73	1.34	2.01	2.01
**0.4**	1.59	1.74	4.65	2.95
**0.5**	3.17	3.63	2.00	1.02
**0.6**	1.84	0.65	1.03	1.02
**0.7**	4.06	2.96	1.01	0.08
**0.8**	2.33	0.81	1.31	0.61
**0.9**	3.67	3.04	2.64	2.84
**Mean Results**	**2.63**	**2.02**	**2.09**	**1.50**

**Table 5:** Summary of stage 2 results: mean values of the estimated contact points (in millimeters) and their errors (in millimeters and % with respect to *L*) for all the experiments.

DOF		Azimuthal			Elevation		
$\lambda_{c}$	$l_{c}^{*}$	$\bar{l_{c}}$	$\bar{e_{l_{c}}}$	$\frac{\bar{e_{l_{c}}}}{L}$(%)	$\bar{l_{c}}$	$\bar{e_{l_{c}}}$	$\frac{\bar{e_{l_{c}}}}{L}$(%)
**0.3**	142.5	153.90	11.40	2.40	153.90	11.40	2.40
**0.4**	190	196.18	6.18	1.30	196.18	6.18	1.30
**0.5**	237.5	245.10	7.60	1.60	245.10	7.60	1.60
**0.6**	285	288.80	3.80	0.80	293.55	8.55	1.80
**0.7**	332.5	333.45	0.95	0.20	335.67	3.16	0.67
**0.8**	380	371.45	−8.55	1.80	371.93	−8.08	1.70
**0.9**	427.5	409.89	−17.51	3.69	409.82	−18.64	3.92
**Mean errors**			**8.00**	**1.68**		**9.09**	**1.91**

**Table 6:** Summary of stage 3 results (I): mean of the settling time responses $t_{s}$ of the outer loop in seconds.

DOF	Azimuthal			Elevation		
$\lambda_{c}$	$|F^{*}|$ (N)			$|F^{*}|$ (N)		
	**0.05**	**0.10**	**0.15**	**0.05**	**0.10**	**0.15**
**0.3**	0.53	0.42	0.27	0.49	0.50	0.34
**0.4**	0.53	0.42	0.29	0.50	0.43	0.28
**0.5**	0.52	0.36	0.18	0.53	0.32	0.20
**0.6**	0.50	0.22	0.24	0.52	0.24	0.19
**0.7**	0.47	0.18	0.29	0.51	0.20	0.37
**0.8**	0.42	0.34	0.47	0.38	0.24	0.40
**0.9**	0.22	0.44	0.54	0.34	0.44	0.51
**Mean Results**	**0.37 s**			**0.38 s**		

**Figure A1:**
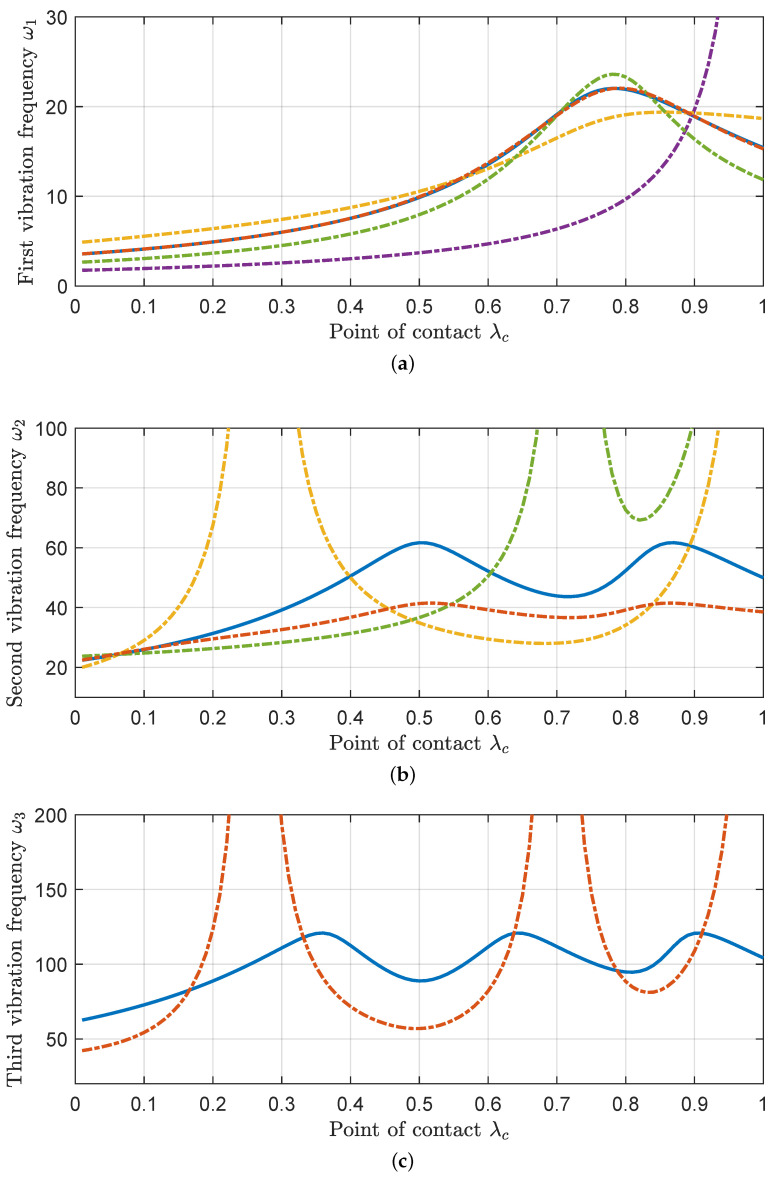
Vibration frequencies as a function of the contact point, where (**a**) shows the first frequency, (**b**) the second, and (**c**) the third. Here (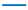) corresponds to the model of [25], (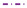) to the model with n=1 masses, (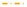) and (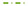) to n=2 masses for $\lambda_{\mu_{1}}=0.27$ and $\lambda_{\mu_{1}}=0.72$ respectively, and (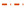) to the model with n=3 masses.

## Data Availability

The dataset is available on request from the authors.

## Abbreviations

The following abbreviations are used in this manuscript:

MSE	Mean square error
RMSE	Root mean square error
F−T	Force−torque
FFT	Fast Fourier Transform
DOF	Degree of freedom

## Appendix A

This appendix presents the derivation of the polynomial coefficients $\upsilon_{i,r}\left(\tau \right)$ of the deflection (11), which are obtained from conditions (12)–(14).

The most immediate coefficient to obtain is $\upsilon_{i,3}\left(\tau \right)$, since taking the fourth derivative of the deflection and applying the last equation of (14) gives

(A1) $$\upsilon_{i,3}\left(\tau \right)=\upsilon_{i+1,3}\left(\tau \right)+\frac{1}{6}{ϝ}_{i}\left(\tau \right)$$

which can be expressed as the following summation

(A2) $$\upsilon_{i,3}\left(\tau \right)=\upsilon_{N,3}\left(\tau \right)+\frac{1}{6}\sum_{p=i}^{N-1}{ϝ}_{p}\left(\tau \right)$$

with $\upsilon_{N,3}\left(\tau \right)=\frac{1}{6}{ϝ}_{N}\left(\tau \right)$ (obtained from (13)). Finally, one arrives at the following result:

(A3) $$\upsilon_{i,3}\left(\tau \right)=\frac{1}{6}\sum_{p=i}^{N}{ϝ}_{p}\left(\tau \right)$$

The coefficient $\upsilon_{i,2}\left(\tau \right)$ is derived from the third equation of (14) as

(A4) $$\upsilon_{i,2}\left(\tau \right)=\upsilon_{i+1,2}\left(\tau \right)-\frac{6}{2}\upsilon_{i,3}\left(\tau \right)\left(\lambda_{i}-\lambda_{i-1}\right)$$

which can also be expressed as a summation of the form

(A5) $$\upsilon_{i,2}\left(\tau \right)=\upsilon_{N,2}\left(\tau \right)-\frac{6}{2}\sum_{k=i}^{N-1}\upsilon_{k,3}\left(\tau \right)\left(\lambda_{k}-\lambda_{k-1}\right)$$

where the value of $\upsilon_{N,2}\left(\tau \right)$ is obtained from (13) such that

(A6) $$\upsilon_{N,2}\left(\tau \right)=-\frac{6}{2}\upsilon_{N,3}\left(\tau \right)\left(\lambda_{N}-\lambda_{N-1}\right)$$

Substituting (A3) into (A5) yields

(A7) $$\upsilon_{i,2}\left(\tau \right)=\upsilon_{N,2}\left(\tau \right)-\frac{1}{2}\sum_{k=i}^{N-1}\sum_{p=k}^{N}{ϝ}_{p}\left(\tau \right)\left(\lambda_{k}-\lambda_{k-1}\right)$$

and interchanging the order of the summations becomes

(A8) $$\upsilon_{i,2}\left(\tau \right)=\upsilon_{N,2}\left(\tau \right)-\frac{1}{2}\sum_{p=i}^{N-1}{ϝ}_{p}\left(\tau \right)\sum_{k=i}^{p}\left(\lambda_{k}-\lambda_{k-1}\right)-\frac{1}{2}{ϝ}_{N}\left(\tau \right)\sum_{k=i}^{N-1}\left(\lambda_{k}-\lambda_{k-1}\right)$$

The telescoping sums of (A8) are developed and used in conjunction with the value of $\upsilon_{N,2}\left(\tau \right)=-\frac{1}{2}{ϝ}_{N}\left(\tau \right)\left(\lambda_{N}-\lambda_{N-1}\right)$ to yield the following result

(A9) $$\upsilon_{i,2}\left(\tau \right)=-\frac{1}{2}\sum_{p=i}^{N}{ϝ}_{p}\left(\tau \right)\left(\lambda_{p}-\lambda_{i-1}\right)$$

The coefficient $\upsilon_{i,1}\left(\tau \right)$ is expressed as

(A10) $$\upsilon_{i,1}\left(\tau \right)=\upsilon_{i-1,1}\left(\tau \right)+2\upsilon_{i-1,2}\left(\tau \right)\left(\lambda_{i-1}-\lambda_{i-2}\right)+3\upsilon_{i-1,3}\left(\tau \right){\left(\lambda_{i-1}-\lambda_{i-2}\right)}^{2}$$

by means of the second equation of (14), and can be reformulated as

(A11) $$\upsilon_{i,1}\left(\tau \right)=\upsilon_{1,1}\left(\tau \right)+2\sum_{k=1}^{i-1}\upsilon_{k,2}\left(\tau \right)\left(\lambda_{k}-\lambda_{k-1}\right)+3\sum_{k=1}^{i-1}\upsilon_{k,3}\left(\tau \right){\left(\lambda_{k}-\lambda_{k-1}\right)}^{2}$$

In this case, the value of $\upsilon_{1,1}\left(\tau \right)=0$ is obtained from Equation (12). Consequently, the application of (A3) and (A9) to (A11) yields the result

(A12) $$\upsilon_{i,1}\left(\tau \right)=\upsilon_{1,1}\left(\tau \right)-\frac{1}{2}\sum_{k=1}^{i-1}\sum_{p=k}^{N}{ϝ}_{p}\left(\tau \right)\left(2\left(\lambda_{p}-\lambda_{k-1}\right)\left(\lambda_{k}-\lambda_{k-1}\right)-{\left(\lambda_{k}-\lambda_{k-1}\right)}^{2}\right)$$

where the summations can be separated as

(A13) $$\begin{array}{c} \upsilon_{i,1}\left(\tau \right)=\upsilon_{1,1}\left(\tau \right)-\frac{1}{2}\sum_{k=1}^{i-1}\sum_{p=k}^{i-1}{ϝ}_{p}\left(\tau \right)\left(2\lambda_{p}\left(\lambda_{k}-\lambda_{k-1}\right)-\left(\lambda_{k}+\lambda_{k-1}\right)\left(\lambda_{k}-\lambda_{k-1}\right)\right)\\ -\frac{1}{2}\sum_{k=1}^{i-1}\sum_{p=i}^{N}{ϝ}_{p}\left(\tau \right)\left(2\lambda_{p}\left(\lambda_{k}-\lambda_{k-1}\right)-\left(\lambda_{k}+\lambda_{k-1}\right)\left(\lambda_{k}-\lambda_{k-1}\right)\right) \end{array}$$

Thus, it is possible to exchange the order of the summations as

(A14) $$\begin{array}{c} \upsilon_{i,1}\left(\tau \right)=\upsilon_{1,1}\left(\tau \right)-\frac{1}{2}\sum_{p=1}^{i-1}{ϝ}_{p}\left(\tau \right)\left(2\lambda_{p}\sum_{k=1}^{p}\left(\lambda_{k}-\lambda_{k-1}\right)-\sum_{k=1}^{p}\left(\lambda_{k}^{2}-\lambda_{k-1}^{2}\right)\right)\\ -\frac{1}{2}\sum_{p=i}^{N}{ϝ}_{p}\left(\tau \right)\left(2\lambda_{p}\sum_{k=1}^{i-1}\left(\lambda_{k}-\lambda_{k-1}\right)-\sum_{k=1}^{i-1}\left(\lambda_{k}^{2}-\lambda_{k-1}^{2}\right)\right) \end{array}$$

By developing the telescopic sums and considering that $\lambda_{0}=0$, we obtain

(A15) $$\upsilon_{i,1}\left(\tau \right)=-\frac{1}{2}\left(\sum_{p=1}^{i-1}{ϝ}_{p}\left(\tau \right)\lambda_{p}^{2}+\sum_{p=i}^{N}{ϝ}_{p}\left(\tau \right)\lambda_{i-1}\left(2\lambda_{p}-\lambda_{i-1}\right)\right)$$

Lastly, the coefficient $\upsilon_{i,0}\left(\tau \right)$ takes the form

(A16) $$\upsilon_{i,0}\left(\tau \right)=\upsilon_{i-1,0}\left(\tau \right)+\upsilon_{i-1,1}\left(\tau \right)\left(\lambda_{i-1}-\lambda_{i-2}\right)+\upsilon_{i-1,2}\left(\tau \right){\left(\lambda_{i-1}-\lambda_{i-2}\right)}^{2}+\upsilon_{i-1,3}\left(\tau \right){\left(\lambda_{i-1}-\lambda_{i-2}\right)}^{3}$$

or, equivalently,

(A17) $$\upsilon_{i,0}\left(\tau \right)=\upsilon_{1,0}\left(\tau \right)+\sum_{k=1}^{i-1}\upsilon_{k,1}\left(\tau \right)\left(\lambda_{k}-\lambda_{k-1}\right)+\sum_{k=1}^{i-1}\upsilon_{k,2}\left(\tau \right){\left(\lambda_{k}-\lambda_{k-1}\right)}^{2}+\sum_{k=1}^{i-1}\upsilon_{k,3}\left(\tau \right){\left(\lambda_{k}-\lambda_{k-1}\right)}^{3}$$

As previously carried out for $\upsilon_{1,1}\left(\tau \right)$, the value of $\upsilon_{1,0}\left(\tau \right)=0$ is obtained from Equation (12). Here, the solution of the previous coefficients is substituted, resulting in the expression

(A18) $$\upsilon_{i,0}\left(\tau \right)=\upsilon_{1,0}\left(\tau \right)-\frac{1}{6}\sum_{k=1}^{i-1}\left(\sum_{p=1}^{k-1}{ϝ}_{p}\left(\tau \right)3\lambda_{p}^{2}\left(\lambda_{k}-\lambda_{k-1}\right)+\sum_{p=k}^{N}{ϝ}_{p}\left(\tau \right)\left(3\lambda_{p}\left(\lambda_{k}^{2}-\lambda_{k-1}^{2}\right)-\left(\lambda_{k}^{3}-\lambda_{k-1}^{3}\right)\right)\right)$$

The summations are separated again as follows:

(A19) $$\begin{array}{c} \upsilon_{i,0}\left(\tau \right)=\upsilon_{1,0}\left(\tau \right)-\frac{1}{6}\left(\sum_{k=2}^{i-1}\sum_{p=1}^{k-1}{ϝ}_{p}\left(\tau \right)3\lambda_{p}^{2}\left(\lambda_{k}-\lambda_{k-1}\right)+\sum_{k=1}^{i-1}\sum_{p=k}^{i-1}{ϝ}_{p}\left(\tau \right)\left(3\lambda_{p}\left(\lambda_{k}^{2}-\lambda_{k-1}^{2}\right)-\left(\lambda_{k}^{3}-\lambda_{k-1}^{3}\right)\right)\right.\\ \left.+\sum_{k=1}^{i-1}\sum_{p=i}^{N}{ϝ}_{p}\left(\tau \right)\left(3\lambda_{p}\left(\lambda_{k}^{2}-\lambda_{k-1}^{2}\right)-\left(\lambda_{k}^{3}-\lambda_{k-1}^{3}\right)\right)\right) \end{array}$$

where it should be noted that i>0 in the first summation. The limit of the first summation is then changed, resulting in the expression

(A20) $$\begin{array}{c} \upsilon_{i,0}\left(\tau \right)=\upsilon_{1,0}\left(\tau \right)-\frac{1}{6}\left(\sum_{k=1}^{i-2}\sum_{p=1}^{k}{ϝ}_{p}\left(\tau \right)3\lambda_{p}^{2}\left(\lambda_{k+1}-\lambda_{k}\right)+\sum_{k=1}^{i-1}\sum_{p=k}^{i-1}{ϝ}_{p}\left(\tau \right)\left(3\lambda_{p}\left(\lambda_{k}^{2}-\lambda_{k-1}^{2}\right)-\left(\lambda_{k}^{3}-\lambda_{k-1}^{3}\right)\right)\right.\\ \left.+\sum_{k=1}^{i-1}\sum_{p=i}^{N}{ϝ}_{p}\left(\tau \right)\left(3\lambda_{p}\left(\lambda_{k}^{2}-\lambda_{k-1}^{2}\right)-\left(\lambda_{k}^{3}-\lambda_{k-1}^{3}\right)\right)\right) \end{array}$$

Subsequently, the order of the summations is exchanged as follows:

(A21) $$\begin{array}{c} \upsilon_{i,0}\left(\tau \right)=\upsilon_{1,0}\left(\tau \right)-\frac{1}{6}\left(\sum_{p=1}^{i-2}{ϝ}_{p}\left(\tau \right)3\lambda_{p}^{2}\sum_{k=p}^{i-2}\left(\lambda_{k+1}-\lambda_{k}\right)+\sum_{p=1}^{i-1}{ϝ}_{p}\left(\tau \right)\left(3\lambda_{p}\sum_{k=1}^{p}\left(\lambda_{k}^{2}-\lambda_{k-1}^{2}\right)-\sum_{k=1}^{p}\left(\lambda_{k}^{3}-\lambda_{k-1}^{3}\right)\right)\right.\\ \left.+\sum_{p=i}^{N}{ϝ}_{p}\left(\tau \right)\left(3\lambda_{p}\sum_{k=1}^{i-1}\left(\lambda_{k}^{2}-\lambda_{k-1}^{2}\right)-\sum_{k=1}^{i-1}\left(\lambda_{k}^{3}-\lambda_{k-1}^{3}\right)\right)\right) \end{array}$$

Finally, by manipulating this equation, we obtain

(A22) $$\upsilon_{i,0}\left(\tau \right)=-\frac{1}{6}\left(\sum_{p=1}^{i-2}{ϝ}_{p}\left(\tau \right)3\lambda_{p}^{2}\left(\lambda_{i-1}-\lambda_{p}\right)+\sum_{p=1}^{i-1}{ϝ}_{p}\left(\tau \right)2\lambda_{p}^{3}+\sum_{p=i}^{N}{ϝ}_{p}\left(\tau \right)\left(\lambda_{i-1}^{2}\left(3\lambda_{p}-\lambda_{i-1}\right)\right)\right)$$

which can be reformulated as

(A23) $$\upsilon_{i,0}\left(\tau \right)=-\frac{1}{6}\left(\sum_{p=1}^{i-2}{ϝ}_{p}\left(\tau \right)\lambda_{p}^{2}\left(3\lambda_{i-1}-\lambda_{p}\right)+{ϝ}_{i-1}\left(\tau \right)2\lambda_{i-1}^{3}+\sum_{p=i}^{N}{ϝ}_{p}\left(\tau \right)\left(\lambda_{i-1}^{2}\left(3\lambda_{p}-\lambda_{i-1}\right)\right)\right)$$

## Appendix B

The results obtained in the frequency fitting for models with a low number of masses (up to n=3 masses) are shown below.

As mentioned above, the model with n=1 masses is imposed by the conservation of mass and length. Therefore $\mu_{1}=1$ and $\lambda_{\mu_{1}}=1$. This model has only one vibration frequency, shown in Figure A1a, and this frequency tends to infinity as the contact approaches the tip of the beam, i.e., the position of the mass.

In the case of the model with n=2 masses, the minimization of (66) is performed by varying $\mu_{1}$ and $\lambda_{\mu_{1}}$ in the interval (0,1) with a step of 0.01. This gives a minimum mean square error of MSE=0.390 for the model whose parameters are $\mu_{1}=0.63$, $\mu_{2}=0.37$, $\lambda_{\mu_{1}}=0.39$, and $\lambda_{\mu_{2}}=1$. However, a local minimum with MSE=0.410 is highlighted for the model with parameters $\mu_{1}=0.63$, $\mu_{2}=0.37$, $\lambda_{\mu_{1}}=0.72$, and $\lambda_{\mu_{2}}=1$ whose MSE is close to the global minimum. These two models have two vibrational frequencies, which are shown in Figure A1a,b. In this case, it is the second frequency that tends to infinity when the contact coincides with one of the masses.

Finally, the model that minimizes the MSE with n=3 masses is shown above, i.e, $\mu_{1}=0.49$, $\mu_{2}=0.41$, $\mu_{3}=0.10$, $\lambda_{\mu_{1}}=0.26$, $\lambda_{\mu_{2}}=0.70$, and $\lambda_{\mu_{3}}=1$ with MSE=0.008. Figure A1 shows the three frequencies of the model and, as in the previous cases, the third frequency tends to infinity when contact occurs at the position of one of the masses.

## Appendix C

The coefficients of the transfer function (72) are adjusted using the Curve Fitting Toolbox in Matlab. For this purpose, the transfer function is obtained by considering that the position of the contact $\lambda_{c}$ changes within the interval (0,1] with a step of 0.001. The following functions are obtained using the robust nonlinear least-squares fitting method with bisquare weights

(A24) $$a\left(\lambda_{c}\right)=\sum_{i=1}^{3}a_{1,i}\exp\left(-{\left(\frac{{\hat{\lambda }}_{c}-a_{2,i}}{a_{3,i}}\right)}^{2}\right)$$

(A25) $$b\left(\lambda_{c}\right)=\sum_{i=1}^{4}b_{1,i}\exp\left(-{\left(\frac{{\hat{\lambda }}_{c}-b_{2,i}}{b_{3,i}}\right)}^{2}\right)$$

(A26) $$c\left(\lambda_{c}\right)=\frac{\sum_{i=0}^{4}c_{1,i}{\hat{\lambda }}_{c}^{i}}{\sum_{i=0}^{4}c_{2,i}{\hat{\lambda }}_{c}^{i}}$$

(A27) $$d\left(\lambda_{c}\right)=d_{1}\lambda_{c}^{d_{2}}+d_{3}$$

where ${\hat{\lambda }}_{c}=\frac{\lambda_{c}-0.5005}{0.2888}$. Figure A2 shows the result of the fit, whereas Table A1 shows the root mean square error RMSE of the fitted functions, and Table A2, Table A3, Table A4 and Table A5 show the coefficients of the functions and their 95% confidence intervals.

**Table A1:** Root mean square error of fitted functions.

	$a(\lambda_{c})$	$b(\lambda_{c})$	$c(\lambda_{c})$	$d(\lambda_{c})$
RMSE	0.7192	2.5815	0.0014	0.1944

**Table A2:** Coefficients (with 95% confidence bounds) of $a(\lambda_{c})$.

*i*	$a_{1,i}$	$a_{2,i}$	$a_{3,i}$
1	137.0 (134.8, 139.2)	0.9716 (0.9708, 0.9725)	0.3589 (0.3565, 0.3612)
2	107.3 (104.2, 110.3)	1.778 (1.737, 1.819)	2.327 (2.305, 2.349)
3	255.4 (253.2, 257.6)	1.068 (1.064, 1.071)	0.7814 (0.7752, 0.7876)

**Table A3:** Coefficients (with 95% confidence bounds) of $b(\lambda_{c})$.

*i*	$b_{1,i}$	$b_{2,i}$	$b_{3,i}$
1	−1669 (−1725, −1612)	1.235 (1.228, 1.242)	0.4159 (0.4133, 0.4185)
2	343.6 (129.5, 557.6)	0.8713 (0.8584, 0.8843)	0.1881 (0.1726, 0.2037)
3	−5364 (−7473, −3254)	5.185 (4.693, 5.676)	2.316 (2.187, 2.444)
4	−1405 (−1591, −1219)	1.051 (1.031, 1.07)	0.2495 (0.2348, 0.2642)

**Table A4:** Coefficients (with 95% confidence bounds) of $c(\lambda_{c})$.

*i*	$c_{1,i}$	$c_{2,i}$
0	−0.2268 (−0.2332, −0.2204)	2.930 (2.851, 3.009)
1	0.7453 (0.7286, 0.7621)	−7.350 (−7.490, −7.210)
2	−0.8945 (−0.9084, −0.8806)	7.529 (7.440, 7.618)
3	0.6424 (0.636, 0.6488)	−3.926 (−3.956, −3.895)
4	−0.3171 (−0.3207, −0.3136)	1 (-)

**Table A5:** Coefficients (with 95% confidence bounds) of $d(\lambda_{c})$.

$d_{1}$	$d_{2}$	$d_{3}$
0.1865 (0.1865, 0.1865)	−2.022 (−2.022, −2.022)	12.64 (12.62, 12.65)
